# Nonlinear mixed selectivity supports reliable neural computation

**DOI:** 10.1371/journal.pcbi.1007544

**Published:** 2020-02-18

**Authors:** W. Jeffrey Johnston, Stephanie E. Palmer, David J. Freedman

**Affiliations:** 1 Graduate Program in Computational Neuroscience, The University of Chicago, Chicago, Illinois, United States of America; 2 Department of Neurobiology, The University of Chicago, Chicago, Illinois, United States of America; 3 Grossman Institute for Neuroscience, Quantitative Biology, and Human Behavior, The University of Chicago, Chicago, Illinois, United States of America; 4 Department of Organismal Biology and Anatomy, The University of Chicago, Chicago, Illinois, United States of America; 5 Department of Physics, The University of Chicago, Chicago, Illinois, United States of America; Columbia University, UNITED STATES

## Abstract

Neuronal activity in the brain is variable, yet both perception and behavior are generally reliable. How does the brain achieve this? Here, we show that the conjunctive coding of multiple stimulus features, commonly known as nonlinear mixed selectivity, may be used by the brain to support reliable information transmission using unreliable neurons. Nonlinearly mixed feature representations have been observed throughout primary sensory, decision-making, and motor brain areas. In these areas, different features are almost always nonlinearly mixed to some degree, rather than represented separately or with only additive (linear) mixing, which we refer to as pure selectivity. Mixed selectivity has been previously shown to support flexible linear decoding for complex behavioral tasks. Here, we show that it has another important benefit: in many cases, it makes orders of magnitude fewer decoding errors than pure selectivity even when both forms of selectivity use the same number of spikes. This benefit holds for sensory, motor, and more abstract, cognitive representations. Further, we show experimental evidence that mixed selectivity exists in the brain even when it does not enable behaviorally useful linear decoding. This suggests that nonlinear mixed selectivity may be a general coding scheme exploited by the brain for reliable and efficient neural computation.

## Author summary

Neurons in the brain are unreliable, while both perception and behavior are generally reliable. In this work, we study how the neural population response to sensory, motor, and cognitive features can produce this reliability. Across the brain, single neurons have been shown to respond to particular conjunctions of multiple features, termed nonlinear mixed selectivity. In this work, we show that populations of these mixed selective neurons lead to many fewer decoding errors than populations without mixed selectivity, even when both neural codes are given the same number of spikes. We show that the reliability benefits from mixed selectivity are quite general, holding under different assumptions about metabolic costs and neural noise as well as for both categorical and sensory errors. Further, previous theoretical work has shown that mixed selectivity enables the learning of complex behaviors with simple decoders. Through the analysis of neural data, we show that the brain implements mixed selectivity even when it would not serve this purpose. Thus, we argue that the brain also implements mixed selectivity to exploit its general benefits for reliable and efficient neural computation.

## Introduction

To support behavior, the brain must use a communication strategy that transmits information about the world faithfully, efficiently, and, perhaps most of all, reliably. The first two of these goals have received extensive attention in neuroscience, particularly in the literature on efficient coding and redundancy reduction [1]. Efficient coding focuses on discovering the response field (RF) for a single neuron that simultaneously maximizes the amount of stimulus information transmitted by the neuron while minimizing the number of spikes that the neuron must fire [1]. A crucial step to this process is representing stimuli without any of the redundancy inherent to the natural world—that is, by isolating and representing the independent components of natural stimuli [2]. Refinements of efficient coding [3] have also emphasized the need for the representation of these components to be neatly packaged, or formatted, so that they are accessible to decoding (as with nonlinear mixed selectivity [4]) and facilitate generalization [5]. As a whole, the ideas of efficient coding have been used to accurately predict the structure of RFs in primary visual cortex [6, 7], and other sensory systems [8–10]. However, existing work on efficient coding, redundancy reduction, and neat packaging primarily addresses the goals of faithful representation and metabolic efficiency. This work does not typically characterize the reliability of decoding after these efficient representations are corrupted by the noise that is inherent to single neuron responses [11, 12]. In fact, non-redundant representations are often highly vulnerable to noise [13].

Making efficient representations robust to the noise present throughout neural systems has received considerably less attention in neuroscience. In information theory, noise robustness is the goal of channel coding. The channel code re-encodes efficient and non-redundant stimulus representations to include redundancy that will increase the robustness of that stimulus representation to later corruption by noise. Recent work has shown that grid cell RFs [14] and the working memory system [15] may implement near-optimal channel codes. In sensory systems, channel coding has been explored more obliquely. Extensive work has focused on deriving RF properties that maximize mutual information between the stimulus and the response [16–19] or the Fisher information from the response function [20–22] (and see [23, 24] for connections between these approaches). However, these measures do not always imply a particular level of decoder performance. Mutual information connects to decoder performance via the rate-distortion bound [25], but different codes with the same mutual information can have different levels of decoder performance relative to that bound [26] (and see S4B Fig). Further, the kind of information encoded matters: a code that has lots of information about a target stimulus without information about which stimuli are nearby to that target will minimize the probability of decoding error, but have worse performance on distance-based measures of error because its errors will be random with respect to the original target; a different code with the same amount of mutual information may make the opposite tradeoff, and minimize distance-based errors while increasing the overall frequency of errors. Evaluating mutual information for each code will not indicate which kind of errors it is likely to make—here, we explore the tradeoff between these two kinds of errors explicitly (see Fig 3). Finally, Fisher information is linked to decoder performance via the Cramer-Rao bound, but saturation of this bound is only guaranteed in low-noise conditions [27] (and codes with less Fisher information can outperform codes with more Fisher information when optimal decoding cannot achieve the bound [28]). There is neural and behavioral [29] evidence that the brain computes successfully on short (e.g., ∼80 ms) timescales and spiking responses have been shown to be highly variable on that timescale [29], thus it is unlikely that the brain typically operates in a low-noise regime.

Here, we analyze an ubiquitous coding strategy in the brain—conjunctive coding for multiple stimulus features—in terms of both its reliability and efficiency. Previous work on conjunctive coding (commonly called nonlinear mixed selectivity [4, 30]) has shown that it produces a neatly packaged and sparse representation that enables the use of simple linear decoders for complex cognitive tasks [4], particularly in the macaque prefrontal cortex [30]. Further, random conjunctive coding has been shown to increase the number of discrete stimuli that can be reliably represented in a neural population [31, 32], particularly in the context of the olfactory system [33–35]; however, a detailed analysis of how the error rate of these codes depends on metabolic cost was not performed. In our work, we develop a novel generalization of nonlinear mixed selectivity, allowing different levels of mixing between stimulus features while preserving full coverage of the stimulus space (see Definition of the codes in Methods). Using these codes, we show that the encoding of stimuli with at least some level of nonlinear mixing almost always produces more reliable and efficient communication than without mixing. Further, we demonstrate novel tradeoffs between codes with and without mixed selectivity—including an analysis of how RF size and error-type affect the optimal level of mixing. Finally, we link our work to experimental data by showing that mixed selectivity is implemented in the brain even when it does not support the flexible linear decoding of stimulus features, but would still play a role in improving the overall reliability of decoding. Our work illustrates that nonlinear mixed selectivity provides highly general benefits to coding reliability and efficiency, and helps to explain the ubiquity of mixed selectivity within sensory [36–41], frontal [4, 30], and motor cortices [42–44].

## Results

### Increased mixing increases stimulus discriminability

**Fig 1:**
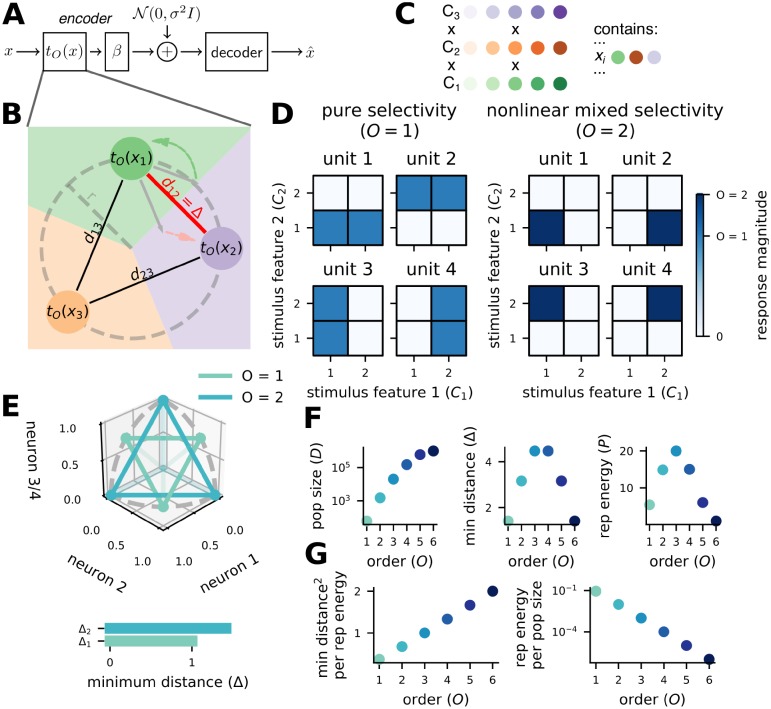
Mixed codes produce more discriminable stimulus representations. **A** The noisy channel model. A stimulus *x* is encoded by encoding function *t*_*O*_(*x*) of order *O*. Next, the linear transform *β* is applied before independent Gaussian-distributed noise with variance *σ*^2^ is added to the representation. Finally, a decoder produces an estimate $\hat{x}$ of the original stimulus. **B** We analyze the encoding function with respect to three important code properties. The minimum distance Δ = *d*_12_ is the smallest distance between any pair of encoded stimuli (codewords), and half of that distance is the nearest border of the Voronoi diagram (background shading). Thus, minimum distance can be used to approximate the probability of decoding error. Representation energy *P* = *r*^2^ is the square of the radius of the circle that all of the codewords lie on. All of the codewords lie in a 2-dimensional plane, so the code has population size *D* = 2. **C** Stimuli are described by *K* features *C*_*i*_ which each take on |*C*_*i*_| = *n* values. All possible combinations of feature values exist, so there are *n*^*K*^ unique stimuli. **D** In pure selectivity (left), units in the code, or neurons, respond to a particular value of one feature and are invariant to changes in other features. In nonlinear mixed selectivity (right), neurons respond to particular combinations of feature values, and the number of feature values in those combinations is defined as the order *O* of the code (here, *O* = 2). **E** The same *O* = 1 and *O* = 2 code as in **D**. (top) The colored points are the response patterns in 3D response space for three of the four neurons in each code. The dashed grey line is the radius of the unit circle centered on the origin for each plane—the two codes are given constant representation energy, and all response patterns lie on the unit 4D hypersphere. For ease of visualization, the vertical dimension in the plot represents both the third and fourth neurons in the population to show three representations from the *O* = 1 code, this does not change the minimum distance. (bottom) The response patterns for the *O* = 2 mixed code have greater minimum distance than those for the *O* = 1 pure code. **F** We derive closed-form expressions for each code metric, and plots of each metric are shown for codes of order 1 to 6 with *K* = 6 and *n* = 10. **G** Mixed codes produce a higher minimum distance per unit representation energy (left) and have a smaller amount of representation energy per neuron than pure codes.

In the brain, stimulus representations are corrupted by noise as they are transmitted between different neural populations. This process can be formalized as transmission down a noisy channel (Fig 1A). The reliability and efficiency of these transmissions depends on the format of the encoded representations—here, we show how three different properties of this representation are affected by nonlinear mixing, and how those properties interact with transmission reliability and efficiency. The three properties of neural representations that we focus on are: minimum distance, neural population size, and metabolic representation energy (Fig 1B and Code properties in Methods).

Minimum distance is the distance between the representations of the two stimuli that are most difficult to discriminate—i.e., that have the most similar neural responses. Importantly, half of this minimum distance represents the smallest magnitude of noise that could cause a decoding error given optimal decoding. Since smaller noise perturbations are more likely to occur than larger ones, errors that map the response to one of the stimuli at minimum distance are more likely than errors to any other stimuli. As a consequence of this, a larger minimum distance typically implies a lower overall probability of error and the minimum distance can be used to develop an accurate approximation of the overall probability of error in many conditions. We develop both a minimum distance-based approximation and an approximation based on the likelihood of all possible errors in Estimating the error rate in Methods. In general, the minimum distance is a more useful metric for summarizing our codes than, for instance, the average distance, because the error rate is a nonlinear function of the distances between individual stimuli. As an example, a code with half of its stimuli at a small minimum distance and the other half of its stimuli at a much further maximum distance would, in most cases, have a much higher error rate than a code that has all of its stimuli at the average of the near and far distances. Population size is the minimum number of independent coding units, or neurons, required to implement the code such that all possible stimuli have a unique response pattern. Representation energy is the metabolic energy consumed by the response of the neural population to a stimulus, defined as the square of the distance between the zero-activity state and the response patterns to stimuli for the code—here, representation energy can be viewed as the squared spike rate in response to a particular stimulus summed across the population of neurons used by the code (though we also consider the sum spike rate, see S1 Fig). In the codes we consider here, all of the stimuli evoke the same number of spikes across the population, and therefore have the same representation energy. Representation energy represents the active, metabolic cost of the code (in terms of the cost of emitting spikes), while population size represents the passive metabolic cost of the code (in terms of neuronal maintenance costs across the population, spiking or not). We begin by considering representation energy alone before considering both together.

The stimuli represented by our codes are described by *K* features that each take one of *n* discrete values (Fig 1C and see Definition of the stimuli in Methods). As a simple example, one feature could be shape, and two values for shape could be square or triangle; a second feature could be color, and two values could be red or blue. In all, there are *n*^*K*^ possible stimuli. So, there are four stimuli in our example. For each stimulus, the likelihood of making an error is the same (see Definition of the stimuli in Methods). In that way, the results we describe here do not depend on the distribution of stimuli. As an example, even if red squares were far more likely to occur than any of the other three stimuli, the error rate would be the same if they were all equally probable. However, in the case where red squares are far more likely than the other stimuli, it would be possible to design a code that dedicates more resources to discriminating red squares than to discriminating the less probable stimuli that would potentially have superior performance to the codes that we study here. We discuss this possibility further in the Discussion and Linear transform (*β*) in Methods. Finally, while we focus on discrete features, our core results are the same with continuous features (see Error-reduction by mixed selectivity in the continuous case in S1 Text).

To understand how mixed selectivity affects code reliability and efficiency, we compare the performance of codes with different levels of conjunctive stimulus feature mixing, following the definition of nonlinear mixed selectivity used previously in the literature [4, 30]. We refer to these different levels of mixing as the order of the neural code. In particular, neurons in a code of order *O* respond to one particular combination of *O* feature values and do not respond otherwise (Fig 1D and see Definition of the codes in Methods), and a code has a neuron that responds to each possible combination of *O* different stimulus feature values (see Code example in Methods for more details). In our example, an order one (*O* = 1) code would have neurons that respond to each shape regardless of color and each color regardless of shape while an order two code (*O* = 2) would have neurons that respond to each combination of shape and color—for instance, one neuron would respond only to red squares, another only to blue squares, and so on. This example can map onto the two features used in the illustration in Fig 1D and 1E. From this construction, each stimulus will have a unique response pattern across the population of neurons, but the population size will vary across code order. In general, higher-order codes will have larger population sizes, but less activity on average per neuron in the population.

With this formalization, we derive closed-form expressions for the population size (*D*_*O*_), representation energy (*P*_*O*_), and minimum distance (Δ_*O*_) of our codes. These expressions are each functions of the number of features *K*, the number of values each of those features can take on *n*, and the order of the code *O* (Fig 1F). The population size for a code of order *O*, with *K* features that each take on *n* values is given by

DO=(KO)nO (1)

which can be viewed in terms of (KO) subpopulations that each encode all possible value combinations of *O* features, *n*^*O*^. Following from this intuition, the representation energy for a code of order *O* is given by

PO=(KO) (2)

That is, there is one neuron active in each of the (KO) subpopulations described above (see Representation energy (*P*_*O*_) of the codes in Methods for more details). Finally, the minimum distance between responses in one of our codes is the distance between the responses to pairs of stimuli that differ only by one feature (though, in *O* = *K* codes, pairs of stimuli that differ by more than one feature are also separated by the minimum distance, see Eq (6) and Code neighbors in S1 Text). Intuitively, this distance is related to the number of neurons active for one stimulus, but not the other. The expression for minimum distance is

ΔO=[2(K-1O-1)]12 (3)

Here, (K-1O-1) gives the number of subpopulations that have different activity when one feature is changed, and the rest of the expression converts that number to the distance between the two representations. We go into more detail on each of these expressions in Code properties in Methods.

Using these expressions, we show that the ratio between squared minimum distance Δ_*O*_ and representation energy *P*_*O*_ is strictly increasing with order for all choices of *K* and *n* (see Minimum distance-representation energy ratio in Methods and Fig 1G, left):

ΔO2PO=2(K-1O-1)(KO) (4)

$$=2\frac{O}{K}$$ (5)

This shows that, given the same amount of representation energy, codes with more mixing produce stimulus representations with strictly larger minimum separation in the response space (Fig 1G, left). Further, higher order codes also have a strictly lower amount of representation energy per neuron in the population (Fig 1G, right).

This increased separation between response patterns with increased code order results from the increased effective dimensionality of the response space of those codes. By effective dimensionality, we mean the smallest number of basis vectors (i.e., dimensions) necessary for a faithful linear reconstruction of the response space—this is equivalent to the number of non-zero eigenvalues in principal components analysis [45]. The effective dimensionality is always less than or equal to the population size of our codes. Intuitively, the higher the effective dimension of the response space, the more response patterns can be arranged within it at a particular distance from each other given the same amount of representation energy. So, codes with higher effective dimensionality will usually have higher minimum distance. To illustrate this, we consider the *O* = *K* case: Here, each stimulus is projected onto its own dimension in the response space. As a result, each stimulus representation will be at the maximum possible distance from all other stimulus representations, assuming only positive responses. If there were fewer effective dimensions than stimuli, it would no longer be possible to place all of the representations at this maximal distance from each other—so, minimum distance is necessarily decreased. Additionally, in the *O* = *K* case, where the response dimension for each stimulus corresponds to a single neuron in the population, this leads to a hyper-sparse representation of the stimuli, where only one neuron fires for each stimulus. However, the same distance between stimulus representations is achieved for any rotation of the response dimensions relative to the neural population. This kind of rotation can be used to produce neural representations that match the heterogeneity of responses of real neurons, in which firing rates are both increased and decreased from the mean response (for an example, see Fig 4C). In particular, instead of hyper-sparse stimulus representations with a single active neuron each, the code can be rotated by the linear transform such that, in an extreme example, each stimulus is represented by a random Gaussian vector, in which almost all of the neurons in the population modulate their firing, and can therefore be considered active, in response to each stimulus (see Linear transform (*β*) in Methods for further discussion). Thus, it is not the sparsity per se that implies these effects, but the higher dimensionality of more, relative to less, mixed codes. While we believe that this distinction is conceptually important, the neural circuit implementation of codes with these rotations is likely to be more involved than for those without, and could have consequences for the efficiency and biological feasibility of these rotated codes.

In practice, the rotation of the response space relative to the neural population can be achieved by the application of a linear transform to the response patterns of our codes (Fig 1A, *β*). In addition to altering the sparsity of neural responses for our codes via rotation, the linear transform can be used to rescale the representation energy used by each code without rotation. In the rest of the text, we only use the linear transform step of encoding to equate the representation energy of codes with different levels of mixing. The linear transform can also be used to expand the population size used by a code—and to exchange few neurons with high individual signal-to-noise ratios (SNRs) for many neurons with lower SNRs and redundant or partially redundant feature tuning. For instance, in our framework, one neuron firing ten spikes in response to a particular stimulus has the same representation energy and distances between stimulus representation as a code that replaces that neuron with two neurons that each fire approximately seven spikes for the same stimulus (due to our squared distance metric for representation energy; our core result holds for a sum of spikes metric as well, see S1 Fig and Sum of spikes representation energy in S1 Text). As experimentally observed neural populations are often composed of neurons with heterogeneous firing rates and SNRs as well as partially redundant feature tuning (as exemplified below, in Fig 4), the linear transform can be used to make our codes exhibit activity that better matches the activity of real neural data.

Importantly, for independent and identically distributed noise that is applied to each neuron after the linear transform (as primarily studied here, Fig 1A, but see Alternate noise models in S1 Text), the change in representation energy produced by the linear transform affects code performance, while the other manipulations discussed above do not. This is because the linear transforms used here (Linear transform (*β*) in Methods) cannot increase or decrease the effective dimensionality of the codes and do not change the underlying relative geometry of the stimulus response patterns to each other except by a uniform scaling. Instead, it is the nonlinear, conjunctive encoding of mixed codes that increases their effective dimensionality, and which produces their greater separation of stimuli in the response space given the same amount of representation energy as pure codes. Due to this, we only use the linear transform to rescale representation energy in the following results.

As a result of their increased separation, mixed codes provide a benefit to decoding for many different noise distributions and decoders (including linear and maximum likelihood decoders), and indicates that mixed codes are likely to produce more reliable and efficient representations than pure codes in a wide variety of conditions. However, to directly quantify transmission reliability (i.e., the probability of a decoding error), we must include the details of both the noise and the decoder (see Fig 1A and Full channel details in Methods).

### Mixed codes make fewer errors than pure codes

To directly estimate the probability of decoding error, or error rate, for each of our codes, we expand our analysis from the encoding function (Fig 1) to the channel as a whole. We choose the noise to be additive, independent, and Gaussian (though we also consider two kinds of Poisson-distributed noise, which give similar results, see S2 Fig and Alternate noise models in S1 Text). Finally, for decoding, we use a maximum likelihood decoder (MLD; and see Full channel details in Methods). Given these noise and decoder assumptions, we can estimate the error rate by decomposing the probability that we make an error into the sum of the probabilities of only the most likely errors (that is, errors to stimuli at minimum distance; see Estimating the error rate in Methods). To proceed with our estimate, we first need to know how many stimulus representations are at minimum distance from a given stimulus for each code, which we refer to as neighbors at minimum distance or nearest neighbors. For stimuli with *K* features that each take on *n* values encoded by a code that conjunctively mixes every combination of *O* features, this is given by,

$$N_{\Delta }\left(O\right)=\{\begin{array}{cc} K(n-1) & O<K\\ n^{K}-1 & O=K \end{array}$$ (6)

To obtain this expression, we show that the distance between stimulus pairs that differ in only one feature is strictly smaller than between those that differ by two features for all codes with *O* < *K*. That is, only pairs of stimuli that differ by a single feature will be at minimum distance from each other, all other pairs of stimuli will be separated by a larger distance. Since there are *K*(*n* − 1) stimuli that differ from each stimulus by one feature, that is the number of neighbors each stimulus has at minimum distance for all codes *O* < *K*. For the *O* = *K* code, since (KK)=1, the code can be viewed as having a single subpopulation. That subpopulation will have different activity for every other stimulus, no matter how many features those other stimuli differ by. Thus, all *n*^*K*^ − 1 other stimuli are at minimum distance from a particular stimulus. We derive these expressions more formally in Code neighbors in S1 Text.

Now, given the number of neighbors at minimum distance, the minimum distance itself, and the assumption of additive Gaussian noise, our estimate of the error rate (PE) takes the following form:

$$PE\approx N_{\Delta }\left(O\right)\;Q(\frac{\Delta_{O}}{2\sqrt{P_{O}}}\text{SNR})$$ (7)

$$=N_{\Delta }\left(O\right)\;Q(\frac{\text{SNR}}{\sqrt{2K/O}})$$ (8)

where *Q*(*y*) is the complementary cumulative distribution function of the standard normal distribution at *y*, $\text{SNR}=\sqrt{V/\sigma^{2}}$ is the population signal-to-noise ratio (see Linear transform (*β*) in Methods), and *N*_Δ_(*O*) is the number of neighbors at minimum distance for the code of order *O*, defined above. This estimate reveals that, for the same SNR, increasing the order of our codes will strictly decrease the probability of a decoding error for codes with order *O* < *K*. In Estimating the error rate in Methods, we use a more detailed estimate to show that the *O* = *K* code will have an even lower error rate than any code with *O* < *K*. That is, the *O* = *K* code will always be the most efficient and robust, given this method of accounting for representation energy and metabolic cost.

**Fig 2:**
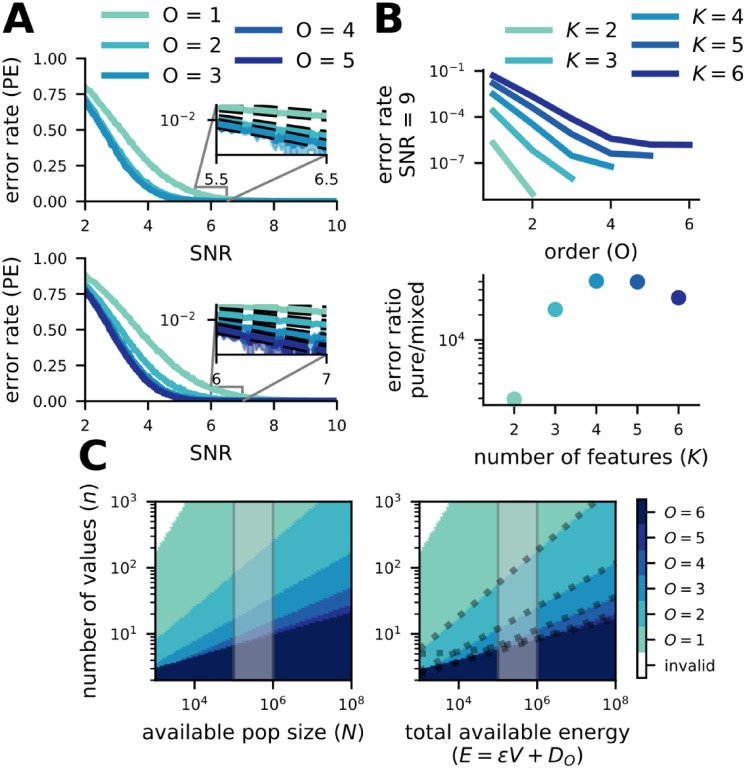
Mixed codes make fewer errors than pure codes. **A** (top) Simulation of codes with *O* = 1, 2, 3 for *K* = 3 and *n* = 5. (inset) For high SNR, code performance is well-approximated by our estimate of error rate. (bottom) Same as above, except with *K* = 5 and *n* = 3. **B** (top) The estimated error rate at a fixed, high SNR (SNR = 9) for codes of every order given a variety of different *K* (all with *n* = 5). Error probability decreases with code order for all codes except, in some cases, the *O* = *K* code. (bottom) The number of errors made by the pure code for every error made by the optimal mixed code at SNR = 9 (as above). In all cases, pure codes make several orders of magnitude more errors than the optimal mixed code. **C** (left) Given a pool of neurons with fixed size, the color corresponding to the code producing the highest minimum distance is shown in the heat map. The shaded area delineates the order of magnitude of the number of neurons believed to be contained in 1 mm^3^ of mouse cortex. (right) The same as on the left, but instead of a pool of neurons of fixed size, each code is given a fixed total amount of energy. The energy is allocated to both passive maintenance of a neural population (with size equal to the population size of the code) and representation energy (increasing SNR). The shaded area is the same as on the left. The dashed lines are plots of our analytical solution for the transition point between the *O* and *O* + 1-order code (see Total energy in Methods).

To verify that our estimate of the error rate is accurate, we numerically simulate codes of all possible orders over a wide range of SNRs for particular choices of *K* and *n* using the same channel as in our analysis. Our simulations show that higher-order codes outperform lower-order codes across all SNRs at which the codes are not saturated at chance or at zero error (Fig 2A). We also show that the estimate closely follows performance for large SNRs (Fig 2A insets). Using this estimate, we compare the error rate of different codes at fixed, high SNR (Fig 2B) and show that pure codes make several orders of magnitude more errors than the mixed code with the optimal order. This also illustrates that, for larger *K*, the full-order mixed code (*O* = *K*) and close to full-order codes (*O* close to *K*) have similar performance (Fig 2B). Due to their smaller population sizes, codes with less than the full amount of mixing (order near *K*) may be desirable in some cases. We make this intuition explicit by accounting for the metabolic cost of neural population size in the following section. In all conditions we simulated (in agreement with our estimate), the fully mixed (*O* = *K*) code had the lowest error rate at a given SNR, though other highly mixed codes (*O* near *K*) reached nearly equivalent error rates with larger numbers of features (*K*; Fig 2A, bottom). Thus, in these conditions, mixed codes provide a significant benefit to coding reliability independent of particular parameter choices.

For smaller choices of *K* and *n*, we were able to empirically evaluate how decoding error compares to the rate-distortion bound [25]. In this context, the rate-distortion bound is an absolute lower bound on the probability of making a decoding error given a particular information rate through the channel (i.e., the mutual information $I(X;\hat{X})$; see The rate-distortion bound and mutual information calculation in S1 Text and S4 Fig). We first show that higher-order codes generate a higher information rate than lower-order codes at most SNRs (S4B Fig, inset)—that is, they more efficiently transform the input into stimulus information. Next, we show that the full-order code (*O* = *K* = 3) fully saturates the rate-distortion bound (S4B Fig). Thus, for a given amount of stimulus information, full-order codes produce as few errors as would be possible for any code [25]. While the *O* = *K* − 1 = 2 code comes close to this bound as well, the pure code does not.

In the above, we have focused on the case with noise applied only to the neural responses in our codes; however, we also show how our codes are affected by two kinds of noise applied to the input stimulus representation (see Alternate noise models in S1 Text). While input noise is often impossible to correct completely [46], we show that input noise that perturbs the input to nearby stimulus values does not affect codes of different orders differently. That is, even though such noise is uncorrectable by our codes, it leads to the same error rate for codes with all levels of mixing (S3A and S3B Fig). Next, we show that input noise that is not confined to perturbations to nearby stimulus values does differentially affect codes of different orders, and can lead to higher error rates in some more mixed codes (S3C Fig). However, we also show that mixed codes still outperform pure codes in many cases due to their greater robustness to output noise (S3D Fig and Fig 2).

### Mixed codes provide benefits despite requiring more neurons

Our analysis so far has focused on the metabolic cost of neuronal spiking. A single spike is thought to be the largest individual metabolic cost in the brain [47]. For a fixed population size *N*, from Eq (5), we know that the code with the highest order *O* such that *D*_*O*_ ≤ *N* will provide the largest minimum distance, given a fixed amount of spiking activity (Fig 2C, left). For a wide range of stimulus set sizes, mixed codes have population sizes less than or equal to an order-of-magnitude estimate of the neuron count in 1 mm^3^ of mouse cortex [48] (Fig 2C, left, shaded region). Thus, the benefits of mixed codes are practically achievable in the brain.

However, the passive maintenance of large neural populations also has a metabolic cost, due to the turnover of ion channels and other cell-level processes [47], which, for large populations of sparsely firing neurons, could be as large if not larger than the metabolic cost associated with spiking. To account for this cost, we adapt the formalization from [49] to relate representation energy (i.e., spiking) to the metabolic cost of population size. We refer to the sum of these costs as the total energy *E* of a code (see Total energy in Methods). Codes with small population sizes will be able to allocate more of their total energy to representation energy, while codes with large population sizes will have less remaining total energy to allocate to representation energy. We do not constrain the maximum SNR that a single neuron in our codes can achieve (even though achievable SNR is limited in the brain [50]). Due to the exponential growth of population size with code order, this choice favors pure codes over mixed codes. In particular, without limiting single neuron SNR, pure codes can allocate the majority of their total energy to the activity of relatively few neurons due to their small population size; if we were to limit single neuron SNR, then pure codes would also have to grow their population size with increased total energy, which would decrease the fraction of that energy used for representation energy. Thus, this analysis serves as a particularly stringent test of the reliability and efficiency of mixed codes.

Mixed codes yield higher minimum distance under the total energy constraint for a wide range of stimulus set sizes and total energy (Fig 2C, right), including order-of-magnitude estimates of the total energy available to 1 mm^3^ of mouse cortex (Fig 2C, right shaded region). Further, our analysis reveals that for any total energy *E* ≥ *n*^2^*K*^2^ (see Eq (M.81)) a mixed code (*O* > 1) will provide better performance than the pure code (*O* = 1). These results also make an important prediction that can be tested experimentally: the order of neuronal RFs should decrease as the fidelity required of the representation increases (i.e., as *n* increases). There already exists indirect experimental support for this prediction. In the visual system, single neurons in primary visual cortex have RFs thought to represent relatively small combinations (small *O*) of low-level stimulus features such as spatial frequency and orientation [6, 36] (but see [51]), while single neurons in the prefrontal cortex are thought to have responses that depend on larger combinations (high *O*) of abstract, often categorical (and therefore low *n*), stimulus features along with behavioral context [4, 30]. However, this pattern has not been rigorously tested, as these regions are rarely recorded in the same tasks and the tasks chosen for each area often follow the form of the prediction—that is, requiring high fidelity (*n*) for investigations of primary sensory areas and low fidelity (*n*) for investigations of prefrontal areas.

### Mixed codes provide reliable coding in sensory systems

**Fig 3:**
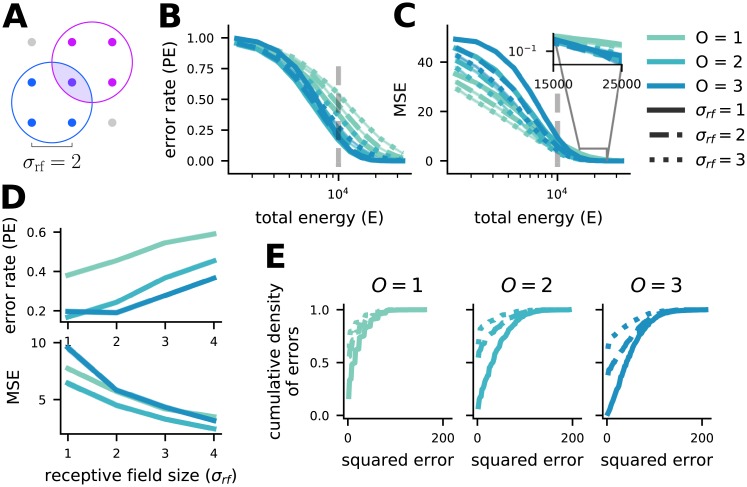
Mixed codes can be more reliable than pure codes for both PE and MSE, but different RF sizes are appropriate for each. **A** An illustration of our RF formalization. With *K* = 2 and *n* = 3, two example RFs of size *σ*_rf_ = 2 are shown. Simultaneous activity from both neurons uniquely specifies the center stimulus point. **B** Simulated PE of codes of all orders for *K* = 3 and *n* = 10 with *σ*_rf_ = 1, 2, 3 (legend as in **C**). Note that total energy is plotted on the x-axis, rather than SNR as in Fig 2. Mixed codes outperform the pure code over many (but not all) total energies. **C** The same as **B** but for MSE rather than PE. Mixed codes perform worse than pure codes for low total energy, but perform better as total energy increases. **D** PE increases (top) and MSE decreases (bottom) as *σ*_rf_ increases for the codes in **B** and **C** taken at the total energy denoted by the dashed grey line. **E** Cumulative distribution functions for the squared errors made by the codes given in **B** and **C** at the grey dashed line. MSE is decreased by increasing *σ*_rf_ despite the increase in PE because the errors that are made become smaller in magnitude and this outweighs their becoming more numerous. This effect is largest for the *O* = *K* = 3 code.

So far, we have focused on the probability of decoding error, which is most applicable to features that represent categorical differences without defined distances from each other (e.g., mistaking a hat for a sock is not clearly less accurate than mistaking a hat for a glove). However, in sensory systems, the features often do have a relational structure and stimuli that are nearby to each other in feature space are also perceptually or semantically similar (e.g., mistaking a 90° orientation for a 180° orientation is clearly less accurate than mistaking 90° for 100°). In the context of sensory information, minimizing the frequency of errors becomes less important than ensuring that the average distance of an estimate from the original stimulus is low. This is because perceptually similar errors are likely more useful for guiding behavior than a random error, even if the latter occurs less frequently. This difference in priority is encapsulated in the contrast between error rate (Fig 3B) and the mean squared-error distortion (MSE; Fig 3C), which is equivalent to the average squared-distance of the estimated stimulus from the original stimulus. In our framework, full-order mixed codes have the highest minimum distance (Eq (5)), but all stimuli are nearest neighbors to all other stimuli (Eq (6)) which causes all errors to be random with respect to the original stimulus. Using MSE instead of error rate, we show that lower-order mixed and pure codes outperform full-order mixed codes at low total energy (Fig 3C). However, increased total energy causes a faster decay in error rates for full-order codes than lower-order codes (Eq (8)), so full-order codes outperform pure codes even under MSE at high total energy (Fig 3B).

Further, experimental investigation of sensory brain regions often reveal neurons with response fields (RFs) that include multiple (sometimes many) perceptually similar stimuli. To investigate how these response fields affect the error rate and MSE of our codes, we generalize our formalization to include RFs (Fig 3A), that can take on different widths, written as *σ*_rf_. Here, instead of responding to a particular combination of *O* feature values, neurons in a code of order *O* will respond when the value of each of *O* features fall within a particular interval of values, with length *σ*_rf_. Thus, each neuron in an order *O* code will respond to a contiguous region of $\sigma_{\text{rf}}^{O}$ stimuli, as illustrated for an *O* = 2 code with *σ*_rf_ = 2 in Fig 3A. This generalization introduces a new dependence of both representation energy and population size on RF width. For representation energy, the generalization is simple: representation energy grows linearly with RF size,

PO=(KO)σrf (9)

because *σ*_rf_ neurons in each population need to be active to unambiguously identify a single size *O* feature-value combination (S6B Fig). For population size, it has previously been shown that increasing RF size can vastly decrease required population size [52, 53]. Here, we find that

DO=(KO)σrf(nσrf+1)O (10)

for *O* > 1, while for *O* = 1 the population size does not change with *σ*_rf_. From this expression, we see that the population size for *O* > 1 codes decreases approximately as $1/\sigma_{\text{rf}}^{O-1}$ with RF size (S6A Fig). Minimum distance does not depend on RF size. In terms of total energy, these dual dependencies on RF size largely cancel each other out, constructing a code with the RF size chosen to minimize total energy consumption does not typically lead to a change in the code order that maximizes minimum distance (S6E and S6G Fig).

Instead, the principal benefit produced by increasing RF size is the reduction of MSE for all codes that results from making errors to nearby stimuli in stimulus space more likely. This is because stimulus decoding now depends on the simultaneous activity of neurons with overlapping RFs, and the most likely errors are now those in which one of that group of neurons is confused for a different neuron that also has an overlapping RF with the rest of the group. Thus, for the full-order code, increasing RF size significantly reduces the randomness of errors and allows the brain to take advantage of their increased minimum distance in the context of sensory systems (Fig 3C). Thus, this work provides a unified framework for understanding the purpose and benefits of large RFs in arbitrary feature spaces, which are often observed in cortex [54]. In particular, increasing RF size decreases the MSE for all codes while increasing the error rate (Fig 3D and see Additional results on response field in S1 Text). The increase in error rate results from the increase in representation energy required for the code without an associated increase in minimum distance; the decrease in MSE results from the fact that the additional representation energy provides information about the stimulus space, which causes errors for larger RF codes to be closer to the original stimulus (Fig 3E). Intuitively, for the the *O* = *K* case, increasing RF size makes stimulus representations non-orthogonal (this can also be viewed as making them less sparse in some conditions), which means that they are no longer positioned at the maximum possible distance from each other (so, the error rate is increased); but, it also means that nearby stimuli now have more similar representations, which makes them more likely errors and leads to the reduction in MSE, correcting the undesirable feature of randomly distributed errors for full-order codes (see Additional results on response field in S1 Text).

We also show increased noise robustness from mixed codes in simulations of a code for continuous stimuli under MSE, using continuous RFs (S7A Fig). Thus, mixed codes are an effective strategy for reliable and efficient coding not just for decision-making systems, but also in sensory systems–which is consistent with their widespread observation in sensory brain regions [36–41].

### Experimental evidence that mixed codes support reliable decoding

Several previous theoretical studies of mixed selectivity have focused on the fact that it enables flexible linear decoding, and there is experimental evidence that the dimensionality expansion provided by mixed selectivity is linked to performance of complex cognitive behaviors [4, 30]. Here, we have shown that mixed codes also provide more general benefits for reliable and efficient information representation in the brain, independent of a particular task and without assuming linear decoding. Thus, our work predicts that mixed codes will be used widely in the brain, instead of being used only for features relevant to particular complex tasks.

**Fig 4:**
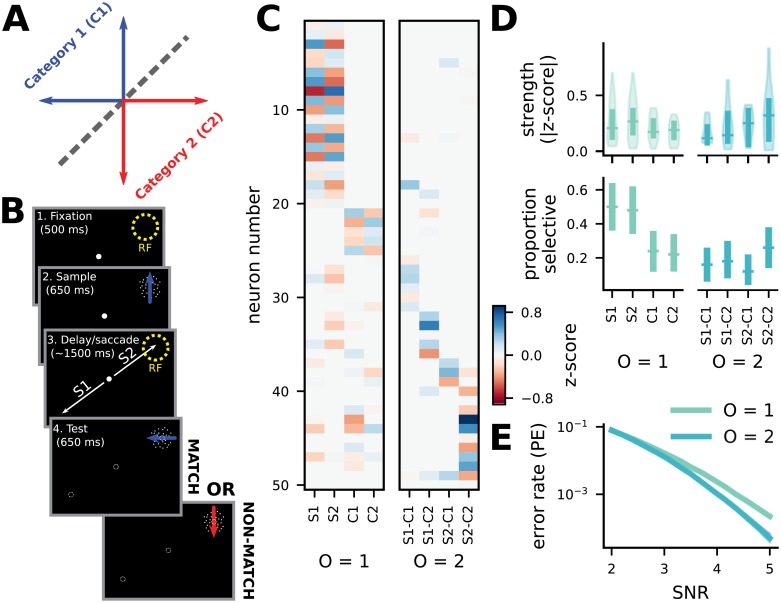
Mixed codes support reliable decoding in the brain, not only flexible computation. **A** The learned, arbitrary category boundary on motion direction used in the saccade DMC task. **B** A schematic of the saccade DMC task. **C** A heatmap of the z-scored magnitude of the coefficients for each term in the linear model. It is sorted by largest magnitude term, from left to right. The linear models were fit using the LASSO method and terms were tested for significance using a permutation test (*p* < .05), only neurons with at least one significant term were included in this and the following plots (50/61 neurons). In addition, 10 out of 71 total recorded neurons were excluded due to having less than 15 recorded trials for at least one condition. **D** (top) The average strength of significant tuning for each term across the neural population, *O* = 1 tuning is on the left, and *O* = 2 tuning is on the right. (bottom) The proportion of neurons in the population that have pure selectivity (left) for the two saccade targets and two categories of motion and nonlinear mixed selectivity (right) for each of the four saccade target and category combinations. Error bars are bootstrapped 95% confidence intervals. **E** Single-feature decoding performance for a code chosen to mirror the conditions of the task, with *K* = 2 and *n* = 2. Mixing features together is advantageous even when decoding those features separately.

To understand whether the brain exploits mixed codes for their general reliability and efficiency rather than only for their ability to enable flexible computation, we test whether the brain implements mixed selectivity when it would not enable the implementation of any behaviorally relevant linear decoders. To do so, we analyze data from a previously published experiment [55] that probed how two behaviorally and semantically independent features are encoded simultaneously by neurons in the lateral intraparietal area (LIP). In the experiment, monkeys performed a delayed match-to-category task in which they were required to categorize a sample visual motion stimulus (Fig 4A), and then remember the sample stimulus category to compare with the category of a test stimulus presented after a delay period (Fig 4B). In addition to the categorization and working memory demands of the task, the animals were also (on some trials) required to make a saccadic eye movement either toward or away from the neuron’s RF during the task’s delay period (Fig 4B, and see Experimental details and task description in Methods). Because LIP activity is known to encode information related to categorical decisions and saccades, this experiment characterized the relationship between the representation of these two features at the single neuron and population level. Despite the saccade being irrelevant to the monkey’s categorical decision in this task, LIP activity demonstrated both pure (*O* = 1, 40/61 neurons were tuned for at least one pure term) and mixed category and saccade tuning (*O* = 2, 31/61 for at least one mixed term; Fig 4C and 4D). This pattern of mixed and pure tuning is consistent with a composite code including RFs of multiple orders. Such codes have performance that falls between codes of either the lowest or highest included order alone, but their heterogeneity may provide other benefits. In particular, a composite *O* = *K* and *O* = *K* − 1 code would have a minimum distance per representation energy ratio between that of each code alone, but would have the same number of nearest neighbors as the *O* = *K* − 1 code (that is, *K*(*n* − 1) rather than *n*^*K*^ − 1 nearest neighbors). Thus, in some cases, this composite code may provide lower MSE distortion than either the *O* = *K* or *O* = *K* − 1 code alone.

Crucially, mixed codes also provide benefits when decoding only one of the two features at a time with a maximum likelihood decoder (Fig 4E). This results from the increased separation between all response patterns produced by mixed codes. However, the same tradeoff that we demonstrated between fully-mixed (*O* = *K*) and less mixed (*O* < *K*) codes for stimulus identity decoding applies to single feature decoding as well. That is, the number of likely errors that result in the decoding of a different feature value is larger for fully-mixed codes than for less-mixed and pure codes, but each of those errors is less likely. To illustrate this, we consider the current case, with *K* = 2 and *n* = 2. Here, for the *O* = 1 code, there is one possible kind of noise perturbation that would lead to an error: one that brings the component corresponding to the correct value of the target feature lower than the component corresponding to the incorrect value of the target feature. Thus, in general, there are *n* − 1 ways to make errors for the *O* = 1 code, and for all the *O* < *K* codes, following from statement 3 in S1 Text. For the *O* = 2 code, there are two kinds of noise perturbations that would cause an error, since there are two components that correspond to the incorrect value of the target feature, rather than just one. In general, there are (*n* − 1)*n*^*K*−1^ ways to make errors for the *O* = *K* code. Thus, we can write an estimate for the single feature decoding error rate that is analogous to Eq (8):

$$PE_{f}\approx N_{\Delta ,f}\left(O\right)\;Q(\frac{\text{SNR}}{\sqrt{2K/O}})$$ (11)

where *PE*_f_ is the probability of making a single feature decoding error and *N*_Δ,f_(*O*) is the number of neighbors with the incorrect feature value that a code of order *O* has at minimum distance. This is written as,

$$N_{\Delta ,f}\left(O\right)=\{\begin{array}{cc} n-1 & O<K\\ \left(n-1\right)n^{K-1} & O=K \end{array}$$ (12)

following from the discussion above. As a result, the utility of mixed codes for single feature decoding is similar to their utility for stimulus identity decoding: For smaller values of *K*, the full-order (*O* = *K*) code will provide the best performance due to its maximal separation of stimulus representations; as *K* grows larger, codes with close to full mixing (*O* near *K*) will begin to all provide equivalent performance.

With two behaviorally and semantically independent features, the brain still implements a mixed code even though it does not enable the implementation of any behaviorally useful linear decoders. The mixed code does, however, improve the reliability and efficiency of the encoding for both features separately and when combined, suggesting that the brain may explicitly utilize mixed codes for that purpose. Further, contemporaneous work has demonstrated that the bat is likely to exploit the reliability benefits of mixed selectivity for the coding of two-dimensional continuous head-direction information—as well as described reliability benefits of full-order mixed codes for continuous stimuli [37] (and see Error-reduction by mixed selectivity in the continuous case in S1 Text).

## Discussion

We have shown that mixed selectivity is an effective and general strategy for reliable and efficient communication. Further, we have demonstrated that, rather than pure (*O* = 1) or fully-mixed (*O* = *K*) codes always providing the most reliable encoding, the optimal code order tends to lie between these two extremes (1 < *O* < *K*) depending on the number of stimulus features, the required fidelity of those features, and the number of neurons or total metabolic energy available to encode the information (Fig 2C). This set of intermediately mixed codes has not previously been analyzed in this context, despite likely being the dominant form of mixed selectivity that exists in the brain. Intermediately mixed codes may also have an important additional benefit. The representations produced by the full-order mixed code (*O* = *K*) in our framework may be difficult to learn and to generalize from [56, 57], due to the fact that each response pattern is the same distance from all other response patterns. Intermediately mixed codes (1 ≤ *O* < *K*) ameliorate this by placing response patterns that are nearby in stimulus space nearby to each other in response space as well. For *O* = *K* codes, we have also shown that nearby stimuli can be placed nearby to each other in response space by increasing RF size, though this increases the representation energy required by the code. This means that intermediately mixed codes and full-order codes with larger RFs carry information not just about the encoded stimulus, but also about which stimuli are nearby to the encoded stimulus, while full-order codes with *σ*_rf_ = 1 carry information only about the encoded stimulus. This information about which stimuli are nearby is likely to be crucial for behavioral performance and learning [58]; however, carrying this extra (from the perspective of stimulus decoding) information often increases the error rate. Lastly, we have shown experimental evidence that the brain implements mixed codes even when they do not facilitate behaviorally relevant linear decoding, but do improve the reliability and efficiency of encoding.

This work differs substantially from most previous work on optimal RFs in four principal ways. First, the dependence of code reliability on RF order, or dimensionality, has not been comprehensively described. While previous work has shown that optimal RF width depends on the dimensionality of the stimulus [20, 59], stimulus dimensionality and RF dimensionality were assumed to be the same. Thus, the effect of changing RF dimensionality in conjunction with RF width was not explored, as it is here (see Fig 3 and S7 Fig). We show that codes using RFs of intermediate dimension (1 < *O* < *K*) are most reliable in a wide variety of cases (Fig 2C), but these codes have not been previously studied outside of binary stimulus features. Second, we directly compute the probability and magnitude of errors for our codes rather than maximizing quantities such as Fisher information and mutual information. This reveals the performance of our codes in low SNR regimes and for different metrics of decoder performance (i.e., error rate and MSE). Third, by accounting for the metabolic cost of both the total spike rate as well as the minimum population size required to implement each of our codes while keeping coverage of the stimulus space constant, we disentangled performance decreases due to a lack of coverage of the stimulus space from those due to the properties of the encoding itself. Fourth, we have investigated differences in code performance across different orders for both discrete and continuous stimuli as well as both binary (error rate) and distance (MSE) error metrics. These different contexts have revealed several nuances, including that, for discrete stimuli, increasing RF size tends to increase error rate, but decrease MSE—highlighting the ways in which RF shape and size can influence which kinds of coding errors are likely for different coding strategies, which has not received extensive study in neuroscience. Thus, this work provides a novel perspective on multiple understudied neural coding problems.

This work also ties directly to existing work in the experimental and theoretical neuroscience literature. Most centrally, we link the previously described flexible linear decoding benefits of mixed selectivity to considerations of reliability and efficiency in neural codes. Experimental work focusing on the utility of mixed selectivity for flexible linear decoding has already demonstrated the ubiquity of mixed codes in prefrontal cortex [4], as well as a putative link from mixed selectivity to behavior [30]. Previous theoretical work has shown that mixed codes with representation rescaling and population expansion can be constructed by the brain naturally in a variety of conditions, due to the nonlinearity of the neuronal input-output function. In particular, this work has demonstrated that random connectivity both in feedforward, binary-thresholded model neurons [31] and in recurrently connected neural network models [56] produces mixed codes for stimulus features. However, randomly constructed networks would require many more neurons than necessary to construct full codes of orders close to *K*. This concern is ameliorated by the learning rules that have been shown to be at work in cortex. Theoretical work that applies biologically plausible, unsupervised Hebbian-like plasticity rules to model networks similar to those in [31, 56] demonstrates that these rules can increase the prevalence of mixed selectivity to levels consistent with those observed experimentally in prefrontal cortex, which has been shown to have more diverse mixed selectivity than expected due to purely random connections[60]. [60]. Thus, not only does this class of mixed codes provide two substantial and separate benefits to the brain (i.e., reliability and linear separability), they are also naturally produced by known neural phenomena—that is, they do not require fine tuning. However, mixed codes with a linear transform that rotates the stimulus representations with respect to the neural population and breaks the link between sparsity and code order, as discussed above and in Linear transform (*β*) in Methods, have a less clear neural circuit implementation. While these codes can replicate the heterogeneity in tuning observed in neural recordings, their implementation is likely to require either extensive nonlinear dendritic processing or recurrent interactions between neurons in the code.

Further, other work in theoretical neuroscience has explored how mixed selectivity can facilitate associative learning [31, 61] as well as negotiate a tradeoff between discrimination and generalization [56]. However, the formalization used in these works differs substantially from the one we use here—and, while our results are broadly consistent with each other (i.e., that the nonlinear dimensionality expansion produced by mixed codes provides benefits to neural computation), these works differ from the current one in the precise level of mixing that they find as optimal. In particular, this previous work finds that lower levels of mixing are optimal, while we show that near-maximal mixing is optimal in our framework in many conditions. Our different conclusions arise from a key difference in our results: in our work, the effective dimension of our codes strictly grows with code order; in the other works, the effective dimension of the codes peaks at relatively low code orders [31, 61]. We believe that this difference arises primarily from two differences in our formalizations. First, we focused on codes with population sizes large enough to implement every combination of inputs at a particular level of mixing—in fact, population size is a function of code order in our framework. The other works, instead, use fixed population sizes across code orders and neurons were given selectivity for random subsets of input combinations. While this may be more biologically plausible in some contexts, previous theoretical work, as discussed above, has shown that biologically plausible plasticity rules can produce mixed selectivity that is significantly different from random, matching patterns observed in cortex [60]. Second, neurons in our codes respond to size *O* conjunctions of particular feature values, which produces a receptive field-like response structure; while, in the other works, neurons respond when the weighted sum of a subset of *O* stimulus feature values exceed a particular threshold, which can produce neurons with more heterogeneous responses. We believe that both of these differences together produce greater dimensionality expansion for large *O* in our framework as compared to the other works, and lead to our different conclusions. While our formalization is, in some ways, less mechanistic than these previous works, we believe that it does more clearly isolate the relative contributions of code order, population size, and representation energy to decoding performance.

Further, interrogation of the bat head-direction system has revealed a dynamic code that appears to shift between mixed and pure representations from moment to moment [37]. The bat head-direction system encodes both the azimuth and pitch of the animal [62], and thus could use either a pure (*O* = 1) or mixed (*O* = 2) code. Surprisingly, the brain appears to use both codes, and adjust the number of neurons with pure or mixed selectivity dynamically depending on the behavioral regime of the animal. In particular, when the animal is maneuvering on short timescales with high angular velocity (i.e., low SNR), the code is biased toward mixed selectivity; and when the animal is navigating over long distances with low angular velocity (i.e., high SNR), the code is biased toward pure selectivity [37]. The authors go on to show that, for a neural population of a size similar to that of the bat head direction system, this dynamic shift is the optimal strategy, as, while the mixed code provides lower decoding error on short timescales, the pure code can produce a finer-grained representation of the full head-direction space and lead to overall smaller errors on long timescales. This work indicates that both mixed and pure codes are decodable by the brain and indicates that the most reliable and efficient code is selected moment-to-moment as the timescale of decoding shifts. This also illustrates an important area for future research in our framework, as the representation energy available to a code is likely to be able to shift from moment-to-moment, while the population size of the code likely cannot. This may have consequences for the optimal code in more dynamic environments. More generally, mixed codes have been observed across diverse sensory and non-sensory systems [4, 30, 36–44, 63], indicating that their usefulness is not only due to enabling flexible linear decoding, but also due to their coding reliability and efficiency.

Our work also points to several areas for future research that will lead to a more detailed characterization of sensory feature representations across different brain regions. First, we have shown that the optimal code order decreases as the fidelity of the stimulus features (i.e., the number of values each feature takes on, *n*) increases. Thus, it will be important to directly compare the fidelity and code order across different levels of the sensory-processing cortical hierarchy. Second, while the benefits of mixed relative to pure codes that we describe here do not rely on a particular stimulus distribution, it is possible that the performance of some codes could be improved by incorporating information about correlations between stimulus features. Intuitively, codes with an order at least the order of the feature correlation structure (e.g., *O* ≥ 2 codes for pairwise feature correlations, *O* ≥ 3 codes for triplet feature correlations, and so on) would be well-suited for this, as the individual SNR of single neurons in the code selective for particular feature combinations could be either increased or decreased if those feature combinations are more or less likely. As an example, if a particular combination of two feature values is more likely than other combinations of those same two features, then any code with *O* ≥ 2 could statically adjust the individual SNR of neurons coding for that feature value combination to maximize performance; but, it would not be possible for an *O* = 1 code to make the same adjustment without assuming a network interaction effect that adjusts the SNR only when the neurons that code for each feature value independently are both active. However, further work is needed to determine whether this strategy improves performance for our codes when accounting for the difference in overall representation energy of the adjusted codes, as well as to determine how the adjustment could be learned in a biologically feasible way.

Overall, our work has shown that mixed selectivity is an effective and practical strategy for reliable coding in the brain. Guaranteeing this reliability, in the face of unreliable neurons, is likely to have fundamentally shaped the functional and even anatomical architecture of neural systems. Developing an understanding of the role of code order, or RF dimensionality, in reliable and efficient coding will give insight into this much broader problem.

## Methods

### Definition of the stimuli

Our stimuli are defined as having *K* features, which each take on a single discrete value. Assuming that our stimuli are discrete simplifies our mathematical analysis, but also makes our analysis relevant to cognitive, categorical representations. In addition, simulations with continuous stimulus features have qualitatively replicated our core results (see Error-reduction by mixed selectivity in the continuous case in S1 Text).

Here, a stimulus is represented by a vector of *K* discrete values. Each value corresponds to one of the *K* features of the stimuli. The nature of the value object does not matter, we only require that it is possible to decide whether two values corresponding to the same feature are equal. For a stimulus *x* with *K* features,

$$x_{i}\in C_{i}$$ (M.1)

for *i* ∈ [1, …, *K*], where *C*_*i*_ is the set (of size *n*_*i*_) of all possible values for feature *i*. Using the equality function, we implement an indicator function,

$$\left[i=j\right]=\{\begin{array}{cc} 0 & i\neq j\\ 1 & i=j \end{array}$$ (M.2)

for all values of all features.

In total, there are *M* = ∏_*i*_ *n*_*i*_ possible stimuli and all of our codes are designed to produce a unique response pattern for each of these *M* possible stimuli. Importantly, our results do not depend on any particular distribution of these stimuli. This follows from statement 4 in S1 Text, which shows that each stimulus has the same set of distances from the other stimuli in code response space. So, all stimuli have the same probability of decoding error—and, thus, heterogeneity in their probability of occurrence will not affect the overall probability of a decoding error.

### Definition of the codes

Our definition for nonlinear mixed selectivity follows that given in [30]. We describe it with some generalizations here.

The codeword *c* corresponding to stimulus *x* ∈ *X* is produced by

$$c\left(x\right)=\beta t_{O}\left(x\right)$$ (M.3)

where *β* is a matrix of size *N* × *D* and *t*_*O*_(*x*) is the encoding function of order *O*. Our codes will primarily be differentiated by *t*_*O*_(*x*), while *β* will be used to equalize their representation energy *V* and population size *N* (see Linear transform (*β*) in Methods).

The elements of the vector *t*_*O*_(*x*) are products of indicator functions, and therefore can only be either one or zero. In particular, for order *O*, the vector *t*_*O*_(*x*) has length corresponding to the number of valid feature-value indicator functions of size *O*—and each element of *t*_*O*_(*x*) corresponds to one of those combinations. More formally, for $A\in G_{K}^{O}$, where $G_{K}^{O}$ is the set of all combinations of *O* elements from [1, …, *K*], and $\left(i,...,j\right)\in \left(C_{A_{1}},...,C_{A_{O}}\right)$,

$$t_{O}{\left(x\right)}_{k}=[x_{A_{1}}=A_{1}^{i}]...[x_{A_{O}}=A_{O}^{j}]$$ (M.4)

with individual neurons (indexed by *k*) corresponding to all feature combinations *A* and all value combinations for those features (*i*, …, *j*).

Thus, 1 ≤ *O* ≤ *K*, where *K* is the total number of stimulus features, and all codes with *O* ≥ 2 are mixed while codes with *O* = 1 are pure codes, following [30]. We will use the term “neuron” to refer to coding units in our models and simulations as well as to refer to biological neurons in the brain to make their analogous roles clear. In our formulation, both mixed and pure codes will always have complete coverage; that is, there will be a neuron coding for every feature value or possible combination of feature values and each of the *M* stimuli will have a corresponding unique codeword.

Finally, in the main text and much of the methods, we will often make the assumption that all stimulus features take on the same number of values—that is, that *n*_*i*_ = *n* for all *i* ∈ [1, …*K*]. While this assumption does change the population size of our codes, it does not change their minimum distance or representation energy (as can be seen below). Thus, it has a negligible effect on our results.

#### Code example

For *K* = 3 and *n* = 2, under our formalization there are codes of three different orders that code for the *n*^*K*^ stimuli.

For *O* = 1, the code has *nK* neurons and below we give some example stimuli (on the left, with the three features each taking on one of their two possible values, 1 or 2) and codewords (across the activity of the neurons, on the right):

111  1 0 1 0 1 0

211  0 1 1 0 1 0

122  1 0 0 1 0 1

222  0 1 0 1 0 1

Note that for each of these stimuli, there are always three neurons responding with 1. Further, the smallest distance between any two codewords is $\sqrt{2}$, between 111 and 211 as well as 122 and 222. This is of course not the smallest number of neurons that we could use to represent the set of 8 stimuli. The smallest number of neurons that could represent these stimuli is log_2_ *n*^*K*^ = log_2_ 8 = 3 neurons, which could use a representation similar to the one we have used to represent the stimuli on the left hand side of the above table.

Thus, this encoding strategy has added redundancy to our representation of the stimuli.

For *O* = 2, the code has (KO)nO=(32)22=12 neurons. It can be viewed as three separate *O* = 2 codes for the three different size 2 subsets of the 3 features. We make that explicit in our example:

111  1 0 0 0  1 0 0 0  1 0 0 0

211  0 1 0 0  1 0 0 0  0 1 0 0

122  0 0 1 0  0 0 0 1  0 0 1 0

222  0 0 0 1  0 0 0 1  0 0 0 1

Note that any two of these three subpopulations alone would produce a code with unique codewords for each of the stimuli. However, they would preferentially represent one of the three features and cause errors to be more likely for the other two features. The minimum distance between any of the stimuli is now 2 and the number of neurons active is 3.

For *O* = 3, the code has *n*^*K*^ = 8 neurons, that each code for a unique combination of the three features—and therefore for a unique stimulus. As in:

111  1 0 0 0 0 0 0 0

211  0 1 0 0 0 0 0 0

122  0 0 0 0 0 0 1 0

222  0 0 0 0 0 0 0 1

Note that there is now only one neuron active for each stimulus, and the minimum distance is $\sqrt{2}$.

Next, we formalize these properties: population size, minimum distance, and representation energy (or the number of active neurons) and derive expressions for each of them for general *K* and *n*.

### Code properties

#### Population size (*D*_*O*_) of the codes

The population size of a code is the length of *t*_*O*_(*x*) for that code. Since we know that a code of order *O* will have an element for each possible combination of feature-values of size *O*, the length of the vector can be framed as a counting problem:

$$D_{O}=\sum_{A\in G_{K}^{O}}\prod_{i\in A}n_{i}$$ (M.5)

where $G_{K}^{O}$ is the set of all subsets of [1, …, *K*] with size *O* and *n*_*i*_ = |*C*_*i*_|. This expression is somewhat cumbersome, so, as described above, we assume that *n* = *n*_*j*_ for all *j* ∈ [1, …, *K*]. This gives,

DO=(KO)nO (M.6)

where (KO) is the binomial coefficient, defined as

(nr)=n!(n-r)!r! (M.7)

if *n* ≥ *r*, otherwise (nr)=0.

For *O* = 1 (the pure code), the population size is

$$D_{1}=Kn$$ (M.8)

and, for *O* = *K* (the fully mixed code), it is

$$D_{K}=n^{K}$$ (M.9)

Thus, the population size (i.e., the length of the vector) grows exponentially with the order of the code.

#### Representation energy (*P*_*O*_) of the codes

We quantify the amount of energy that each coding scheme uses to transmit codewords. In particular, we will model energy in two ways and will see that these are equivalent for large *n*_*i*_ and do not substantially change our results for smaller *n*_*i*_.

In the first way, we take the firing rate variance of each neuron (or code dimension) across the stimulus set as the energy used by that neuron for coding. The sum of all the variances across the population gives the representation energy of the code. With the definition of variance,

$$\text{Var}\left(X\right)=E(X^{2})-E{\left(X\right)}^{2}$$

we can express representation energy (*P*_*O*_) as:

$$P_{O}=\sum_{i}^{D_{O}}\text{Var}{\left(t_{O}{\left(x\right)}_{i}\right)}_{X}$$ (M.10)

$$=\sum_{A\in G_{K}^{O}}\prod_{i\in A}n_{i}[\frac{1}{\prod_{i\in A}n_{i}}-\frac{1}{\prod_{i\in A}n_{i}^{2}}]$$ (M.11)

$$=\sum_{A\in G_{K}^{O}}[1-\frac{1}{\prod_{i\in A}n_{i}}]$$ (M.12)

≤(KO) (M.13)

where $G_{K}^{O}$ is the set of all subsets of size *O* of *K* elements (here, features).

With large *n*_*i*_ the second term in the sum becomes very small, and we can see that the upper bound of the last line gives a good approximation of the representation energy.

So, for *O* = 1,

$$P_{1}\approx K$$ (M.14)

For, *O* = *K*,

$$P_{K}\approx 1$$ (M.15)

In the second way, we notice that, for a code of a particular order, all of the codewords have the same distance from the zero-activity state (the origin). This distance provides a different notion of energy consumption, through $P_{O}=w_{O}^{2}$, where *w*_*O*_ is the distance for a code of order *O*. Formally, it differs from the notion of energy consumption given above only in that the squared mean activity is not subtracted. That is,

$$P_{O}=w_{O}^{2}=\sum_{i}^{D}E{\left(t_{i}{\left(x\right)}^{2}\right)}_{X}$$ (M.16)

rather than

$$P_{O}=\sum_{i}^{D}E{\left(t_{i}{\left(x\right)}^{2}\right)}_{X}-\sum_{i}^{D}E{\left(t_{i}\left(x\right)\right)}_{X}^{2}$$ (M.17)

Following the derivation above, the distance is:

w=[(KO)]12 (M.18)

and the representation energy (or squared distance) is

PO=(KO) (M.19)

and, for *O* = 1,

$$P_{1}=K$$ (M.20)

and, for *O* = *K*,

$$P_{K}=1$$ (M.21)

That is, this gives the same answer as our other measure for energy, but does not depend on the assumption that the *n*_*i*_ are large.

Use of either of these two measures does not substantively affect our results. In our simulations, we will use the former because it slightly benefits pure codes (because the mean activity of neurons in pure codes is generally higher than that in mixed codes, so there is a larger reduction in their representation energy by the subtraction of the squared mean) and we are exploring the benefits of mixed codes.

#### Minimum distance (Δ) of the codes

The smallest distance between any two codewords is directly related to the probability that a decoder will make an error when attempting to discriminate between those two codewords, and can be used to bound the performance of decoders in general.

Intuitively, the minimum distance will be between stimuli that differ by only one feature. In particular, the minimum distance will be dependent on how many of the (KO) combinations of *O* features contain the feature that differs between the two stimuli. This is given by (K-1O-1) (which counts the ways one can select the rest of the *O* − 1 features, assuming the differing feature is already included in the combination), and figures prominently in our equation for minimum distance below. We also develop an equation for the distance between stimuli that differ by more than one feature and show that this distance is increasing in the number of features two stimuli differ by, see Code distances in S1 Text. These two steps show that this intuition about the minimum distance is correct.

By statement 2 in S1 Text, we know that the minimum value of *d*(*K*, *O*, *v*) occurs when *v* = 1. We can then evaluate our expression for distance, found in statement 1 in S1 Text, at *v* = 1

d(K,O,1)=[2∑i1(1i)(K-1O-i)]12 (M.22)

=[2(11)(K-1O-1)]12 (M.23)

ΔO=[2(K-1O-1)]12 (M.24)

Now, we can evaluate this expression for any *K* and *O* that we desire. For *O* = 1,

$$\Delta_{1}=\sqrt{2}$$ (M.25)

and, for *O* = *K*,

$$\Delta_{K}=\sqrt{2}$$ (M.26)

While the minimum distance for these two codes is the same, their representation energy is different (see Eqs (M.14) and (M.15)). Further, minimum distance and power are both weakly unimodal around *O* = ⌊*K*/2⌋ (Fig 1E, center and right).

### Minimum distance-representation energy ratio

A straightforward way to describe code performance in a single number is to take the ratio between squared minimum distance and representation energy. Codes with larger ratios will typically have a lower probability of decoding error given the same noise level.

Δ2P=2(K-1O-1)(KO) (M.27)

$$=2\frac{(K-O)!O!(K-1)!}{(K-O)!(O-1)!K!}$$ (M.28)

$$=2\frac{O}{K}$$ (M.29)

which is strictly increasing with order (Fig 1F, left).

### Linear transform (*β*)

In comparing codes of different orders, it useful to give the codes the same representation energy (*P*_*O*_) and population size (*D*_*O*_), so that it is clear that the differences in performance are due to the different effective dimensionalities and arrangements of stimulus representations produced by codes of different orders, rather than differences in representation energy or population size. Thus, we apply a linear transform *β* to the codewords *t*_*O*_(*x*), which sets representation energy to be equal to some value *V* and population size to be equal to some value *N*, which are both now decoupled from code order, *O*. Thus, we can flexibly compare codes of different orders given the same energy—or, put another way, using *β*, codes of different orders can be implemented with arbitrary representation energy and relatively unconstrained population sizes (though, *D*_*O*_ ≤ *N*). In practice, *β* is an *N* × *D*_*O*_ matrix.

This step also has an important conceptual interpretation. In neural recordings, the activity of large populations of neurons have been found to inhabit a subspace of all possible neural responses with much lower dimensionality than the number of neurons (i.e., the maximum possible dimensionality, if all neurons were independent). In our framework, this subspace can be viewed as the *D*_*O*_-dimensional space of the codewords, while a neural implementation of the code may use *N* neurons (with *N* ≥ *D*_*O*_). Through the transform from the *D*_*O*_-dimensional codeword space to the *N*-dimensional response space, *β* can be used to make several quantitative and qualitative changes to the final representation. We will summarize the ones that are most relevant here:

1. It can expand (*P*_*O*_ < *V*) or contract (*P*_*O*_ > *V*) the representation by either increasing or decreasing the representation energy of the code.
2. It can perform a rotation or reflection of the codewords, which can both change the sparsity of the neural response as well as move from the strict binary representation of the codewords to a more graded representation, where neurons can have different non-zero firing rates for different stimuli. For instance, each element of the linear transform matrix can be sampled from a Normal distribution with zero mean (and normalized according to the conditions set out below). Then, each neuron in the population would have a non-zero response to every stimulus with high probability.
3. It can project the codewords into a higher-dimensional space, as when *D*_*O*_ < *N*—in this way, there can be a tradeoff made between having fewer neurons with high individual SNRs or more neurons with lower individual SNRs, to result in the same population-level SNR (which is the SNR used in the rest of the manuscript). For example, in this way, a single neuron with a high individual SNR could be replaced by two neurons with lower individual SNRs but the same feature tuning without affecting the decoding performance of the code. Thus, the population size N of the code would be larger, but both the effective dimensionality and code performance would not be affected.

Thus, through the linear transform, the population representation of the stimuli can be made more realistic and heterogeneous. In addition, as shown in Fig 1G, the sparsity of our codewords increases with code order. Through choice of *β*, that dependence can be, in part, broken.

Importantly, only the change in representation energy due to the linear transform will alter code performance (see below). Here, we only consider linear transforms that scale all of the codeword dimensions uniformly–and, as a consequence, maintain the uniform representation energy across individual codewords. This class of linear transforms cannot change the relative geometry of the codeword representations, it can only rescale, rotate, and embed it. This relative geometry is what produces the increase in code performance with code order at the same representation energy that we describe here.

Heterogeneous rescaling of the codeword dimensions by either a static or dynamic linear transform is one way in which a particular code could be optimized for the representation of a non-uniform stimulus distribution. We consider this possibility further in the Discussion.

#### Selecting a linear transform (*β*)

In choosing *β*, we must satisfy four constraints:

1. *N* ≥ *D*_*O*_
2. *β*^†^*β* = *I*, where *I* is the *D*_*O*_ × *D*_*O*_ identity matrix and *β*^†^ is the pseudoinverse of *β*.
3. The vector length, *H*, of each column in *β* must be the same.
4. E(*β*_*ij*_*β*_*ik*_)_*j* ≠ *k*_ = 0; this will be true, for instance, for *β* where the rows or columns are sampled from an independent Normal distribution.

Even given these constraints, there is significant flexibility in the choice of *β*, which allows *β* to be used to alter some of the qualitative features of our codes, as described above. However, as mentioned above and as we will show in more detail below, it is only the vector length of the columns of *β*, *H*, that affects the performance of the code. As a result, throughout our simulations, we will use *β*s that are proportional to the identity matrix for simplicity and ease of interpretation.

For *β* with length *H*, the transformed code *βt*_*O*_(*x*), where *t*_*O*_(*x*) has representation energy *P*_*O*_, will have representation energy

$$V=H^{2}P_{O}$$ (M.30)

We derive this using the squared distance definition of energy. So, the energy of the original code *t*_*O*_(*x*) is given by,

PO=(KO)

After applying *β*, we want to find the square of the average distance of the codewords from the origin, or *V*, under the squared distance definition of energy.

So, we want to find, where *c*(*x*) = *βt*_*O*_(*x*), *X* is the set of stimuli, and *M* is the number of stimuli,

$$V=E[\sum_{i}^{N}{\left(c_{i}\left(x\right)\right)}^{2}]$$ (M.31)

$$=\frac{1}{M}\sum_{x\in X}\sum_{i}^{N}{\left(c_{i}\left(x\right)\right)}^{2}$$ (M.32)

$$=\frac{1}{M}\sum_{x\in X}\sum_{i}^{N}{\left(\sum_{j\in D_{x}}\beta_{ij}\right)}^{2}$$ (M.33)

$$\text{where}\;D_{x}\;\text{is}\;\text{the}\;\text{set}\;\text{of}\;\text{non-zero}\;\text{indices}\;\text{of}\;t_{O}\left(x\right)\;\text{for}\;x$$ (M.34)

$$=\frac{1}{M}\sum_{x\in X}\sum_{i}^{N}\sum_{j\in D_{x}}\beta_{ij}^{2}$$ (M.35)

bythedefinitionofβ,constraint4 (M.36)

$$=\frac{1}{M}\sum_{x\in X}\sum_{j\in D_{x}}H^{2}$$ (M.37)

bythedefinitionofβ,constraint3 (M.38)

=1M∑x∈X(KO)H2 (M.39)

=H2(KO) (M.40)

$$=H^{2}P_{O}$$ (M.41)

Thus, we can give different codes the same representation energy *V* by choosing $H=\sqrt{V/P_{O}}$ for each *O*.

#### The effect of *β* on minimum distance

The distance between two points *c*_*i*_ = *βt*_*O*_(*x*_*i*_) and *c*_*j*_ = *βt*_*O*_(*x*_*j*_), represented as $d_{ij}^{\beta }$, is given by

$$d_{ij}^{\beta }=Hd_{ij}$$ (M.42)

where *d*_*ij*_ is the distance between the two points *t*_*O*_(*x*_*i*_) and *t*_*O*_(*x*_*j*_).

We know that points *x*_*i*_ and *x*_*j*_ are both $\sqrt{P}$ units away from the origin while codewords *c*_*i*_ and *c*_*j*_ are $H\sqrt{P}$ units from the origin (by Eqs (M.30) and (M.18)). We want to find $d_{ij}^{\beta }$.

The angle between the two points is

$$\theta =\sin^{-1}\frac{\frac{1}{2}d_{ij}}{\sqrt{P}}$$

so, we can rearrange to find:

$$d_{ij}^{\beta }=2H\sqrt{P}\sin\theta$$ (M.43)

$$=2H\sqrt{P}\frac{\frac{1}{2}d_{ij}}{\sqrt{P}}$$ (M.44)

$$=Hd_{ij}$$ (M.45)

It follows directly from Eq (M.42) that the minimum distance after *β* is applied, *δ*, is given by

δ=HΔ (M.46)

Further, it follows that the ratio given in Eq (M.29) is not altered by *H*, or choice of particular *β*, since

$$\frac{\delta_{O}^{2}}{V_{O}}=\frac{H^{2}\Delta_{O}^{2}}{H^{2}P_{O}}=\frac{\Delta_{O}^{2}}{P_{O}}$$ (M.47)

$$=2\frac{O}{K}$$ (M.48)

### Full channel details

We simulated codes of all possible orders for particular choices of *K* and *n*. Three important choices were made for these simulations. First, the codewords from each code were passed through a linear transform *β*. The linear transform was used to equate the population size and representation energy of different order codes, such that we could investigate code performance when each order of code had the same number of participating units and the same signal-to-noise ratio ($\text{SNR}=\sqrt{V/\sigma^{2}}$ where *V* is the code representation energy after the linear transform is applied and *σ*^2^ is the noise variance), as in Fig 2 and see Linear transform (*β*) in Methods. Second, the noise in the channel was chosen to be additive and to follow an independent Normal distribution across code dimensions. Third, we use maximum likelihood decoding (MLD) to estimate the original stimulus. This choice is consistent with Bayesian and probabilistic formulations of neural encoding and decoding [64–66]. While inclusion of noise correlations would be an interesting topic for future research, we show here that they are not essential for any performance increases due to nonlinear, conjunctive mixing.

#### Code availability

All of the code for the simulations was written in Python (3.6.4) using NumPy (1.14.2), SciPy (1.0.1) [67], and Scikit-learn (0.18.1) [68]. The code is available on github. For each SNR and each code order, 5000 to 10000 trials were simulated.

### Estimating the error rate

While the minimum distance-representation energy ratio we derive in Eq (M.29) provides useful insight into the performance of codes of different orders, it does not give a direct estimate of the probability of decoding error. In particular, it is difficult to interpret the magnitude of performance differences without incorporating the magnitude of the noise itself, the decoder used, and the arrangement of all of the codewords in coding space to estimate error rate directly. Here, we incorporate the details of the full channel to directly estimate the error rate via a union bound estimate (UBE).

That is, with the channel,

r(x)=c(x)+η (M.49)

$$=\beta t_{O}\left(x\right)+\eta$$ (M.50)

where *η* ∼ N(0, *σ*^2^) (see Fig 1A for a schematic) and a maximum likelihood decoding function *f* such that $\hat{x}=f\left(r\left(x\right)\right)$ where $\hat{x}$ is the maximum likelihood estimate of *x* given *r*(*x*), we want to estimate the probability that $\hat{x}\neq x$ across *X*—that is, the probability of decoding error, PE. To begin,

$$\text{PE}=\sum_{x\in X}p\left(x\right)P(\cup_{x\neq a\in X}\hat{X}=a|X=x)$$ (M.51)

$$=P(\cup_{x\neq a\in X}\hat{X}=a|X=x)$$ (M.52)

bystatement4inS1Text (M.53)

$$=\sum_{x\neq a\in X}P\left(\hat{X}=a|X=x\right)$$ (M.54)

bythedisjointnatureofdecodingevents (M.55)

$$\leq \sum_{x\neq a\in X}Q(\frac{d_{E}\left(x,a\right)}{2\sigma })$$ (M.56)

where *Q*(*y*) is the ccdf at *y* of N(0,1) and *d*_E_(*x*, *y*) is the Euclidean distance between the codewords corresponding to *x* and *y* (i.e., the Euclidean distance between *βt*_*O*_(*x*) and *βt*_*O*_(*y*)).

We can proceed further by using the function:

d(K,O,v)=[2∑iv(vi)(K-vO-i)]12 (M.57)

which gives the distance between two stimuli that differ by *v* out of *K* total features in an order *O* code (see Code distances in S1 Text for a derivation), and the fact that the number of stimuli that differ by *v* features from a particular stimulus is given by

Nall(v)=(Kv)(n-1)v (M.58)

Thus, Eq (M.56) can be rewritten as a sum of all stimuli *x* ≠ *a* arranged by their distance from *x* (the original stimulus):

$$PE\leq \sum_{x\neq a\in X}Q(\frac{d_{E}\left(x,a\right)}{2\sigma })=\sum_{v=1}^{K}N_{\text{all}}\left(v\right)\;Q(\frac{Hd(K,O,v)}{2\sigma })$$ (M.59)

$$\text{where}\;H=\sqrt{V/P_{O}}\text{,}\;\text{due}\;\text{to}\;\text{the}\;\text{linear}\;\text{transform}$$ (M.60)

$$=\sum_{v=1}^{K}N_{\text{all}}\left(v\right)\;Q(\sqrt{\frac{V}{P_{O}}}\frac{d(K,O,v)}{2\sigma })$$ (M.61)

$$=\sum_{v=1}^{K}N_{\text{all}}\left(v\right)\;Q(\frac{\text{SNR}}{2\sqrt{P_{O}}}\;d\left(K,O,v\right))$$ (M.62)

This expression provides an explicit upper bound that well-approximates our simulation results (Fig 2A) and we use this expression to characterize code performance in Fig 2B. However, it is difficult to gain intuition about code performance through this expression. Thus, we reformulate the sum to include only the terms that give the likelihood of errors to stimuli at minimum distance. This works as an approximation because these errors require the smallest noise and are therefore, in most cases, exponentially more likely than errors to stimuli at even the next smallest distance. Using our expression for minimum distance and for the number of stimuli at that distance for each code:

$$\text{PE}\leq \sum_{v=1}^{K}N_{\text{all}}\left(v\right)\;Q(\frac{\text{SNR}}{2\sqrt{P_{O}}}\;d\left(K,O,v\right))$$ (M.63)

$$\approx N_{\Delta }\left(O\right)Q(\frac{\text{SNR}}{2\sqrt{P_{O}}}\;d\left(K,O,1\right))$$ (M.64)

$$=N_{\Delta }\left(O\right)Q(\frac{\text{SNR}}{2}\;\frac{\Delta_{O}}{\sqrt{P_{O}}})$$ (M.65)

$$=N_{\Delta }\left(O\right)Q(\frac{\text{SNR}}{\sqrt{2K/O}})$$ (M.66)

where *N*_Δ_(*O*) is the number of neighbors at minimum distance for the code of order *O*, derived in Code neighbors in S1 Text. Thus, we can see that PE depends most strongly on the minimum distance-representation energy ratio and SNR. Further, for *O* < *K*, this approximation is strictly decreasing with order, implying the main result of our paper: that increasing mixing (*O*) increases code reliability. This is matched by our simulation results (Fig 2A). Further, in the full approximation above, the *O* = *K* code is guaranteed to have the smallest error rate, due to the fact that all of its stimulus representations are at maximum distance from each other ($d\left(x,a\right)=\sqrt{2V}$, derived in Code distances in S1 Text), while all other codes have at least some proportion of stimuli that are closer together (and therefore are more likely to give rise to errors). This is also matched by our simulation results (Fig 2A), though, for large *K*, the performance of high-order codes becomes increasingly similar (Fig 2A, bottom and Fig 2B).

### Total energy

Similar to [49], we assume that all neurons, whether spiking or not, consume some baseline, non-zero amount of energy—due to passive maintenance processes, including the circulation of ion channels, and due to spontaneous activity. We define this amount of energy to be equal to one unit. Next, we assume that spiking neurons consume the baseline energy plus an amount of energy proportional to the square of their firing activity; this activity summed across the population is the representation energy (*P*_*O*_). So, the total energy consumption of a code, *E*, can be written:

$$E=\epsilon V+D_{O}$$ (M.67)

where *ϵ* controls the proportional cost of spiking relative to passive maintenance costs. This *ϵ* will vary between neuron types, but has been estimated by experiment to be around 10 to 10^2^ [49].

From Eq (M.67), we see that a code of order *O* allocated *E* total energy would have,

$$V=\frac{E-D_{O}}{\epsilon }$$ (M.68)

and

$$\delta^{2}=\frac{2O}{K\epsilon }(E-D_{O})$$ (M.69)

where only codes with *V* > 0 (that is, *E* > *D*_*O*_) can be implemented in practice. This *δ* is used in the comparisons for Fig 2C. From this expression, we observe that the particular value of *ϵ* does not change the relative performance of codes with different orders. So, our results in Fig 2C do not depend on *ϵ*.

Further, we find when *δ*_*O*_ = *δ*_*O*+1_ as a function of *E* to discover when the *O* + 1-order code will begin to outperform the *O*-order code:

$$\delta_{O}^{2}=\delta_{O+1}^{2}$$ (M.70)

$$\frac{2O}{K\epsilon }(E-D_{O})=\frac{2(O+1)}{K\epsilon }(E-D_{O+1})$$ (M.71)

$$O(E-D_{O})=\left(O+1\right)(E-D_{O+1})$$ (M.72)

$$OE-OD_{O}=\left(O+1\right)E-\left(O+1\right)D_{O+1}$$ (M.73)

$$E=\left(O+1\right)D_{O+1}-OD_{O}$$ (M.74)

=(O+1)(KO+1)nO+1-O(KO)nO (M.75)

=(K-O)(KO)nO+1-O(KO)nO (M.76)

=(K-O)(KO)nO+1-On(KO)nO+1 (M.77)

=nK-(n+1)On(KO)nO+1 (M.78)

=(nK-(n+1)O)(KO)nO (M.79)

$$E_{O\to O+1}=(nK-(n+1)O)D_{O}$$ (M.80)

and using this for *O* = 1, we find that

$$E_{\text{mixed}}=n^{2}K^{2}-n^{2}K-nK$$ (M.81)

$$<n^{2}K^{2}$$ (M.82)

such that for *E* > *E*_mixed_ a mixed code (i.e., a code of order *O* > 1) will always provide better performance than a pure code.

### Experimental details and task description

We used experimental data in Fig 4 that was previously published in a separate study [55]. The full methods are given in the original paper, though we briefly review several key points here. The data may be requested from the authors of the previous study.

#### The behavioral task

See the schematic in Fig 4B. First, a moving dot stimulus in a direction that was on one side of a learned category boundary was presented while the animal fixated. Then, there was a delay period during which the animal was compelled to saccade to one of two locations before, finally, a second motion stimulus was presented and the animal reported whether the category of the first (or sample) stimulus matched the category of the second (or test) stimulus. The division of the 360° of motion direction into two contiguous categories was arbitrary, and learned by the animals over extensive training.

#### The electrophysiological recordings and analysis

The experimenters recorded from 64 lateral intraparietal area (LIP) neurons in two monkeys (monkey J: *n* = 35; monkey M: *n* = 29) during performance of the DMC task. Recordings were performed using single 75 *μ*m tungsten microelectrodes (FHC). Units were sorted offline, and selected for quality and stability. No information about the LIP subdivision from which each neuron was collected is available.

Linear models for motion category (category 1 or 2) and saccade direction (toward or away from the neuronal RF) with interaction terms (between category and saccade direction) were fit using an L1 prior in scikit-learn [68] (i.e., the Lasso fitting procedure) to all neurons with greater than 15 trials for each of the four conditions (61/71 neurons). The data used for fitting was subsampled without replacement so that each condition had the same number of trials as the condition with the fewest recorded trials (e.g., if there were 40, 35, 24, and 37 from each condition for a single neuron, then 24 trials would be subsampled from each group for the fitting). Fit coefficients were tested for significance via a permutation test (using 5,000 permutations) at the *p* < .05 level after applying a Bonferroni correction for multiple comparisons. Spikes were counted in the 20 ms to 170 ms window after the saccade was made and then spike counts for each neuron were z-scored across the four conditions.

## Supporting information

S1 Text Additional results and derivations, including seven supplementary figures. In the supplementary text, we include additional detail on several points discussed in the main text as well as explore several related topics. In particular, we
Provide a glossary of variables used throughout the paper.Derive the general expression for distance between stimulus representation as well as show that it is increasing.Show several important code neighbor properties.Show the consequences of using Manhattan distance for representation energy rather than squared Euclidean distance, as used in the rest of the paper.Explore several alternate noise models, including Poisson-distributed output noise and input noise.Show that full order codes satisfy the rate-distortion bound in some conditions.Demonstrate the representation energy required to reach a particular level of error, related to the results shown in Fig 2.Derive and show additional properties of the response field size manipulation, related to Fig 3.Show that mixed codes reduce errors for continuous stimuli and receptive fields as well.
(PDF)

S1 Fig Using sum-of-spikes instead of squared distance representation energy improves the performance of higher-order codes, related to Fig 2. **A** (top) The minimum distance per representation energy ratio (Δ_*O*_/*P*_*O*_) for distance representation energy; and (bottom) the representation energy per population size ratio (*P*_*O*_/*D*_*O*_). **B** Simulation of codes with *O* = 1, 2, 3, 4 for *K* = 4 and *n* = 4. (inset) Performance of the codes relative to the approximation (dashed lines). **C** (left) Using our approximation, we show that for different *K* (with *n* = 5) the SNR required to reach 0.1% decoding error has its minimum at *O* = *K*. (right) The representation energy required by the pure code relative to that required by the best mixed code (given by point color and label) to reach 0.1% decoding error.
(EPS)

S2 FigChannels with pure Poisson and Poisson-with-baseline noise have similar performance to those with Gaussian noise, related to Fig 2.**A** The error rate (PE) as a function of representation energy (*V*) for codes with pure Poisson distributed noise, *K* = 3 and *n* = 5. **B** The error rate (PE, axis same as on the left) as a function of poisson SNR for codes with Poisson-with-baseline distributed noise. Poisson SNR is defined as $\sqrt{V/r_{\text{spont}}}$, with *K* = 3, *n* = 5, and *r*_spont_ = .2. Representation energy ranges from .2 to 10, as on the left. Low values were chosen for both representation energy and *r*_spont_ to allow an analogue to the binary bit flip case. The gray shaded area is the region where .2 to 3.2 spikes of signal are expected across the population and few neurons will fire more than once.(EPS)

S3 Fig Code order does not have an effect on sensitivity to local input noise, related to Fig 2. For all panels, K = 3, n = 10. **A** The mean squared-error (MSE) of different codes as a function of input noise without output noise, represented as the probability of each feature taking on the value above or below its “true” value. **B** The same as **A** but for the *O* = 3 code with different RF sizes. **C** An additional simulation with non-local input noise—where bits in an input *O* = 1 code are randomly flipped with the probability given on the x-axis. The error rate of the resulting *O* = 1, 2, 3 codes with the same parameters as above is plotted. **D** A simulation with non-local input noise and output noise. The result here is similar to that without input noise in Fig 3, except that the *O* = 3 code has a higher error rate at high SNR due to its increased sensitivity to input noise, shown in **C**.
(EPS)

S4 FigThe mixed codes come close to or achieve the rate-distortion bound while the pure code does not, related to Fig 2.**A** A schematic of the rate-distortion bound. The bound is a function on the information rate-error rate plane dividing a region of possible codes from a region of impossible codes. The bound depends only on the stimulus distribution and distortion type, it does not depend on any code properties. Thus, we evaluate codes relative to the bound. If a code achieves the bound, that means it achieves the most efficient possible mapping from stimulus information to distortion—i.e., it uses the fewest possible bits to achieve a particular error rate. The rate-distortion bound goes to zero as $I(X;\hat{X})$ approaches *H*(*X*) since the mutual information between the stimulus and its estimate cannot exceed the entropy of the stimulus. **B** For *K* = 3, *n* = 5 and a uniform probability distribution over the stimuli, we evaluated codes with different levels of mixing relative to the rate-distortion bound (red). We show that the two mixed codes *O* = 2 and *O* = 3 achieve or come close to achieving the rate-distortion bound, while the pure code does not. (inset) The transformation from SNR to $I(X;\hat{X})$ for each of the codes is fairly similar, though the mixed codes are slightly less efficient at low SNR and slightly more efficient at high SNR.(EPS)

S5 Fig Mixed codes require less representation energy to achieve the same error rates as pure codes, related to Fig 2. For both plots, *n* = 5 and the noise variance *σ*^2^ = 10. **A** The amount of representation energy required to reach a 1% error rate for codes of all orders given various numbers of features *K*. The code requiring the least energy is always the *O* = *K* or *O* = *K* − 1 code. **B** The percent more representation energy required by the pure code to reach a 1% error rate compared to the optimal mixed code. The order of the optimal mixed code is indicated by the text above each marker.
(EPS)

S6 Fig Changing response field (RF) size changes code properties, related to Fig 3. **A** The number of dimensions required to implement the code decreases by several orders of magnitude. **B** The power of the code increases by several orders of magnitude. **C** The tradeoff between minimum distance and code power remains constant if all codes are given the same RF size. **D** The RF size maximizing minimum distance under the total energy constraint differs between codes. **E** The code providing the highest minimum distance with *σ*_rf_ = 1 (left) and *σ*_rf_ = *σ*_rf,opt_ (right) as computed in Eq (S.2). They are only marginally different. **F** The optimal RF size for codes of different orders given features with different numbers of possible values. **G** Histogram of the differences in code order giving the highest distance from **E**.
(EPS)

S7 Fig The benefits of mixed codes broadly generalize to continuous stimuli and RFs, related to Fig 3. **A** The MSE of codes of all orders with *K* = 3. The higher-order codes provide better performance than the lower-order codes. **B** MSE increases with RF size, which is contrary to the result in the discrete case (Fig 3d). **C** The cumulative distribution function of squared error for the three codes and for three different RF sizes.
(EPS)
