# Supplementary material for: Nonlinear mixed selectivity supports reliable neural computation
Source: PLoS Comput Biol. 2020 Feb 18;16(2):e1007544. doi: 10.1371/journal.pcbi.1007544 (PMC7048320; doi:10.1371/journal.pcbi.1007544)
Supplement: S1 Text — In the supplementary text, we include additional detail on several points discussed in the main text as well as explore several related topics. In particular, we Provide a glossary of variables used throughout the paper.Derive the general expression for distance between stimulus representation as well as show that it is increasing.Show several important code neighbor properties.Show the consequences of using Manhattan distance for representation energy rather than squared Euclidean distance, as used in the rest of the paper.Explore several alternate noise models, including Poisson-distributed output noise and input noise.Show that full order codes satisfy the rate-distortion bound in some conditions.Demonstrate the representation energy required to reach a particular level of error, related to the results shown in Fig 2.Derive and show additional properties of the response field size manipulation, related to Fig 3.Show that mixed codes reduce errors for continuous stimuli and receptive fields as well. (PDF) [file pcbi.1007544.s001.pdf]

A Text S1

A.1 Glossary of terms

M	The number of stimuli transmitted by a code.
Δ_O	The minimum distance of the code of order O.
δ_O	The minimum distance of a code of order O after β is applied.
P_O	The representation energy used by a code of order O.
V	The representation energy used by a code after β is applied.
D_O	The population size of a code of order O.
N	The population size of the code after β is applied.
K	The number of features that a stimulus has.
C_i	The set of values that feature i can take on.
n_i	The size of set C_i; that is, $n_i = \|C_i\|$
G_K^s	The set of all possible subsets of $[1, ..., K]$ with size s; $\{X \subset [1, ..., K] : \|X\| = s\}$
x	A stimulus; a vector of length K, where $x_i \in C_i$ for all i.
$t_O(x)$	The encoding function of order O. It takes a stimulus (x) and produces the representation of that stimulus in a code of order O – also referred to as the codeword. The representation is a vector of length D_O of ones and zeros.
β	The amplifying transform. It is applied to the codeword ($t_O(x)$) and produces the amplified encoding; β is a matrix of size $N \times D$ and must satisfy the constraints given in Linear transform (β) in Methods.
H	The power in each column of β; $\sqrt{\sum_i^N \beta_{ij}^2} = H$ for all j.
η	A noise term. Here, always Gaussian, with $\eta \sim \mathrm{N}(0, \sigma^2)$.
$c(x)$	The amplified codeword corresponding to a given stimulus, $c(x) = \beta t_O(x)$. It is a vector of length N.
$r(x)$	The noisy amplified codeword corresponding to a given stimulus, $r(x) = c(x) + \eta$. It is a vector of length N.
$f(r)$	The maximum likelihood decoding function for a particular code. It solves the equation $\mathrm{argmax}_x\, P(r\|x)P(x)/P(r)$.
$\hat{x}$	The estimate of x, derived from a noisy representation, $\hat{x} = f(r)$.

A.2 Code distances

We develop some general properties of the distances between stimulus representations in our codes here. These are useful in conclusively proving the minimum distance, as well as showing that each stimulus has the same neighbor structure as all the other stimuli in a particular code.

Statement 1. *The distance between two stimulus codewords is given by*

$$d(K, O, v) = \left[2 \sum_i^v \binom{v}{i} \binom{K-v}{O-i}\right]^{\frac{1}{2}} \tag{S.1}$$

where v is the number of features the stimuli differ in, O is the order of the code, and K is the number of features.

Derivation. Using the set G_K^O with $|G_K^O| = \binom{K}{O}$, we see that when we change a feature $i \in [1, ..., K]$, by the definition of the indicator function and of our codes, we know that

one term (a product of indicator functions) in each feature combination that includes i will flip from 0 to 1 and another term will flip from 1 to 0. Thus, given the subset $B_i^O = \{b \in G_K^O | i \in b\}$, we obtain a distance of $\sqrt{2|B_i^O|}$ from changing the value of feature i. When we change a second term, j, we obtain $B_j^O = \{b \in G_K^O | j \in b\}$. The distance between the two stimuli is then related to the size of the union of these two sets: $\sqrt{2|B_i^O \cup B_j^O|}$.

So, to find the distance between two codewords, we need to count the number of features in which they differ and then find the distance, given the order of the code O and the number of stimulus features K.

$$d(K,O,v) = \left[2\left|\bigcup_i^v B_i^O\right|\right]^{\frac{1}{2}} \tag{S.2}$$

$$= \left[2\sum_i^v \binom{v}{i}\binom{K-v}{O-i}\right]^{\frac{1}{2}} \tag{S.3}$$

where the second binomial coefficient counts the number of subsets containing exactly i of the v changed features and the first binomial coefficient counts the number of different ways i features could be chosen from the v changed features. Since our codes include all combinations, the identities of the features changed does not matter – only the number of them. □

Next, it will be useful to know that this distance function is increasing with v, as, combined with statement 1, it will allow us to find the minimum distance.

Statement 2. *The function $d(K,O,v)$ is increasing with v.*

Derivation. We want to show that $d(K,O,v) \leq d(K,O,v+1)$.

$$0 \leq d(K,O,v+1)^2 - d(K,O,v)^2 \tag{S.4}$$

$$= \left|\bigcup_i^{v+1} B_i^O\right| - \left|\bigcup_i^v B_i^O\right| \tag{S.5}$$

$$= \left|B_{v+1}^O \setminus \bigcup_i^v B_i^O\right| \tag{S.6}$$

where the last line is the size of the set of values that are in B_{v+1}^O and not in any of the other B_i^O for $i \in [1, ..., v]$. The relationship holds because a set cannot have a negative size. Thus, $d(K,O,v+1) \geq d(K,O,v)$ and therefore the function d is increasing in v. □

Finally, we will derive the maximum distance between any two codewords in a code. Intuitively, this will be when none of the same neurons are active for the two codewords. We can see this from our equations above by noticing that $d(K,O,v)$ has a (potentially

non-unique) maximum at $v = K$ (by statement 2) and

$$\begin{aligned} d(K,O,K) &= \left[2\left|\bigcup_i^K B_i^O\right|\right]^{\frac{1}{2}} && \text{(S.7)} \\ &= \left[2\left|G_K^O\right|\right]^{\frac{1}{2}} && \text{(S.8)} \\ &= \left[2\binom{K}{O}\right]^{\frac{1}{2}} && \text{(S.9)} \\ &= \left[2P_O\right]^{\frac{1}{2}} && \text{(S.10)} \end{aligned}$$

After the linear transform, this becomes $\sqrt{2V}$, and therefore does not depend on code order. Finally, we identify this maximum distance as equivalent to the minimum distance of the $O = K$ code after application of the linear transform:

$$\begin{aligned} \delta_K &= \sqrt{\frac{V}{P_K}}\Delta_K && \text{(S.11)} \\ &= \sqrt{V2\frac{K}{K}} && \text{(S.12)} \\ &\text{by Eq (M.29)} && \text{(S.13)} \\ &= \sqrt{2V} && \text{(S.14)} \end{aligned}$$

which demonstrates that all stimulus representations in the $O = K$ code are at maximum distance from each other, by statement 2.

A.3 Code neighbors

For the UBE, it becomes necessary to know the number of codewords at minimum distance from any given codeword ($N_\Delta(O)$).
Statement 3. *The number of neighbors at minimum distance for a code of order O $N_\Delta(O)$ is given by:*

$$N_\Delta(O) = \begin{cases} K(n-1) & O < K \\ n^K - 1 & O = K \end{cases} \qquad \text{(S.15)}$$

Derivation. From the fact that the distance function is increasing with v (statement 2), we know that $d(K,O,1)$ is the minimum of $d(K,O,v)$, but it may or may not be a unique minimum.

Thus, we want to find O such that $d(K, O, 1) < d(K, O, 2)$,

$$
\begin{aligned}
0 &< d(K,O,2)^2 - d(K,O,1)^2 && \text{(S.16)}\\
&= \binom{2}{2}\binom{K-2}{O-2} + \binom{2}{1}\binom{K-2}{O-1} - \binom{1}{1}\binom{K-1}{O-1} && \text{(S.17)}\\
&= \binom{K-2}{O-2} + 2\binom{K-2}{O-1} - \binom{K-1}{O-1} && \text{(S.18)}\\
&\text{exploiting binomial identities to make all binomial terms equal} && \text{(S.19)}\\
&= \binom{K-2}{O-2} + 2\frac{K-2+1-O+1}{O-1}\binom{K-2}{O-2} - \frac{K-1}{O-1}\binom{K-2}{O-2} && \text{(S.20)}\\
&= \binom{K-2}{O-2} + 2\frac{K-O}{O-1}\binom{K-2}{O-2} - \frac{K-1}{O-1}\binom{K-2}{O-2} && \text{(S.21)}\\
&= \left[1 + 2\frac{K-O}{O-1} - \frac{K-1}{O-1}\right]\binom{K-2}{O-2} && \text{(S.22)}\\
&= \frac{O-1+2K-2O-K+1}{O-1}\binom{K-2}{O-2} && \text{(S.23)}\\
&= \frac{K-O}{O-1}\binom{K-2}{O-2} && \text{(S.24)}\\
&\text{this is undefined for } O = 1\text{, which is undesirable} && \text{(S.25)}\\
&= \frac{K-1}{K-1}\frac{K-O}{O-1}\binom{K-2}{O-2} && \text{(S.26)}\\
0 &< \frac{K-O}{K-1}\binom{K-1}{O-1} && \text{(S.27)}
\end{aligned}
$$

This last expression is true when $1 \leq O < K$ and false otherwise (i.e., when $O = K$). When it is true, it implies that changing one stimulus feature produces codewords at a closer distance than changing two stimulus features. Now, we must find how many stimuli differ by a single feature from a given stimulus. Any single feature of the K features could be changed, and it could be changed to any one of $n - 1$ different values (excluding its current value) – so, $N_\Delta(O) = K(n-1)$ for $O < K$.

If $O = K$, then $G_K^K = \{\{1, ..., K\}\} = B_1^K$ and since B_i^K cannot grow beyond the size of G_K^O, all codewords must be at the same distance. Thus, $N_\Delta(O) = n^K - 1$ for $O = K$. □

Statement 4. *The number of neighbors at a fixed distance does not depend on codeword identity.*

Derivation. We assume that the number of neighbors at a fixed distance does depend on codeword identity and show that this leads to a contradiction. We know that codeword distance does not depend on original codeword identity (statement 1), but does depend on the number of features that the stimuli differ by. Thus, for a set of codewords to have more neighbors at a particular distance than a different set of codewords, the corresponding set of stimuli must be able to differ in more ways from the corresponding set of other stimuli. Stimuli can differ by changing 1 to K of their K features to one of the $n - 1$ different values for each feature C_i. For a set of stimuli to be able to differ in more ways than a different set of stimuli, that set of stimuli must have either more features or more possible values for each feature. Either of these would contradict our definition of the stimuli (see Definition of the stimuli in Methods). □

A.4 Sum of spikes representation energy

To this point, we have used the squared distance or variance to characterize the relationship of spiking activity across the population to metabolic energy consumption in the form of representation energy. This is following decades of literature on neural coding [1] and communication theory [2]. However, there is some evidence to suggest that a sum of spikes, or L1, representation energy metric may be more appropriate for use in the brain [3]. To gain intuition into how this different metric for metabolic energy affects our results, we perform simulations and modify our analytical approximation to use this metric. The relevant approximation is now:

$$\text{PE} \leq \sum_{v=1}^{K} N_{\text{all}}(v)\, Q\left(\frac{V}{P_O}\frac{d(K,O,v)}{2\sigma}\right) \tag{S.28}$$

because $H = V/P_O$ for the linear transform.

These results illustrate that, for large numbers of features K, some intermediately mixed codes, particularly with order close to 1, will provide worse performance than the pure code, but still that highly mixed codes always provide the best performance (see Fig S1). A further consideration of code performance with the L1 norm may be an interesting area for future research.

A.5 Alternate noise models

In the main text, we focus on additive, Gaussian noise. However, multiple other noise models have been proposed to be relevant to the brain, including Poisson, bit-flip, and input noise. We consider all of those briefly here.

A.5.1 Poisson and bit-flip noise

To this point, the noise in our neural channel has been Gaussian distributed, which allows us to vary the SNR down our channel independently of representation energy or firing rate. However, neural firing rates are often viewed, at least roughly, as following a Poisson process, which implies a particular SNR at different firing rates due to a strict relationship between mean firing rate and firing rate variance (though experimentally observed firing rate-SNR relationships have not followed the one expected from a Poisson process [4]). Thus, it is possible that due to the different firing rates of individual neurons used in our codes (as only the sum firing rate is held constant across codes), Poisson noise could change which code performs best.

To address this concern, we perform simulations with Poisson, instead of additive Gaussian, noise, following:

$$r(x) = f\left(\beta t_O(x)\right) \tag{S.29}$$

where $f(x)$ produces a sample from a Poisson distribution with mean x and the linear transform β is proportional to the D_O identity matrix. The results of these simulations are given in Fig S2A. We can see that, in this case, the qualitative performance of our codes relative to each other is not affected – and mixed codes still outperform pure codes. This is expected from previous work.

However, pure Poisson noise, modeled in this way, may not be appropriate for the nervous system. In particular, for our function $f(x)$, where $x = 0$ the result is 0 with

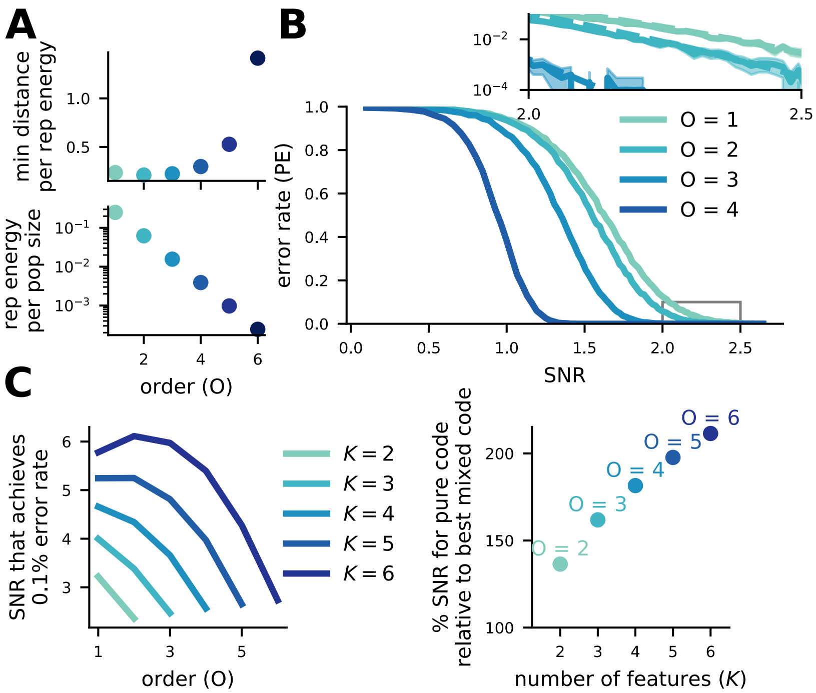

Fig S1. Using sum-of-spikes instead of squared distance representation energy improves the performance of higher-order codes, related to Fig 2. **A** (top) The minimum distance per representation energy ratio (Δ_O/P_O) for distance representation energy; and (bottom) the representation energy per population size ratio (P_O/D_O). **B** Simulation of codes with $O = 1, 2, 3, 4$ for $K = 4$ and $n = 4$. (inset) Performance of the codes relative to the approximation (dashed lines). **C** (left) Using our approximation, we show that for different K (with $n = 5$) the SNR required to reach 0.1 % decoding error has its minimum at $O = K$. (right) The representation energy required by the pure code relative to that required by the best mixed code (given by point color and label) to reach 0.1 % decoding error.

probability 1, as is the case for a Poisson distribution. In contrast, neurons observed in the brain almost always have a non-zero spike probability due to spontaneous activity. To model this spontaneous activity, we include a baseline firing rate in our noise model, taking

$$r(x) = g\left(\beta t_O(x)\right) \tag{S.30}$$

where $g(x) = f\left(\min\left(x, r_{\text{spont}}\right)\right)$, r_{spont} is the spontaneous firing rate in the neural population, and $f(.)$ is defined as above. Thus, all neurons will have a non-zero probability of emitting noise spikes at all representation energies. The result of the simulations for these conditions are given in Fig S2B. Here, mixed codes still tend to perform better than pure codes. However, the $O = K$ mixed code performs worse relative to other mixed codes than with either Gaussian or pure Poisson noise.

For low representation energy (as in the shaded gray area of Fig S2B, where there will be only, on average .2 to 3.2 spikes of signal across the population), these Poisson-with-baseline simulations approximate the conditions of binary bit-flip noise (though the flip probability is not symmetric), and indicate that mixed codes outperform pure codes in those conditions as well.

In summary, the pattern of our results holds for numerous different response noise

(i.e., channel-noise) distributions. This underlines the generality of the results derived from our three code metrics.

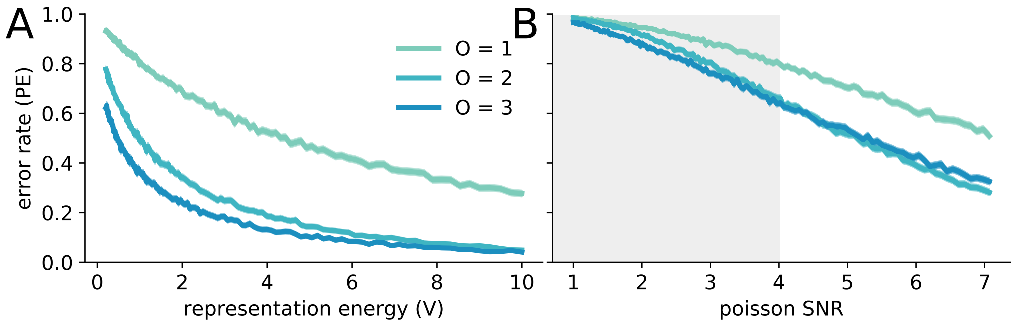

Fig S2. Channels with pure Poisson and Poisson-with-baseline noise have similar performance to those with Gaussian noise, related to Fig 2. **A** The error rate (PE) as a function of representation energy (V) for codes with pure Poisson distributed noise, $K = 3$ and $n = 5$. **B** The error rate (PE, axis same as on the left) as a function of poisson SNR for codes with Poisson-with-baseline distributed noise. Poisson SNR is defined as $\sqrt{V/r_{\text{spont}}}$, with $K = 3$, $n = 5$, and $r_{\text{spont}} = .2$. Representation energy ranges from .2 to 10, as on the left. Low values were chosen for both representation energy and r_{spont} to allow an analogue to the binary bit flip case. The gray shaded area is the region where .2 to 3.2 spikes of signal are expected across the population and few neurons will fire more than once.

A.5.2 Input noise

Noise in a neural system affects both the output of, as modeled in the main text and above, and the input to that system. Here, we investigate how input noise affects the robustness of codes with different levels of mixing. Previous work on mixed codes has argued that codes with more mixing are especially sensitive to input noise, and thus may make it difficult to recognize highly mixed representations of similar stimuli as similar to each other [56]. In our framework, it is true that mixed codes map similar stimuli to distant locations in response space (in part, this is what is meant by having large minimum distance, and the discrimination-generalization tradeoff discussed in [56]).

However, when the provided input is either the "true" stimulus or one of the adjacent stimuli in stimulus space (i.e., input noise is local), as would be the case if the input was from a decoder that makes local errors (e.g., with low mean squared-error), code order does not affect robustness to input noise for decoding. This is because, while the input noise can create very different representations in response space for high-order codes, the decoder maps those different representations back to nearby areas of stimulus space, creating errors only as large as the noise in the input. This result is counter to the intuition provided by previous investigations of mixed codes with random stimuli [56]. We illustrate this without any output noise in Fig S3A, B.

We also simulate non-local input noise, where the input is assumed to be an $O = 1$ code stimulus representation that is subject to bit-flip noise. In this case, the $O = K$ code has the highest MSE, as expected from the previous literature, while both $O < K$ codes that we simulated have the same MSE. To explore why this is, we consider the consequences of a single input bit-flip. There are two possibilities:

1. With probability $\frac{K}{Kn} = \frac{1}{n}$, the bit-flip will change a 1 to a 0 for one of the features.

- For an $O = K$ code, this means that none of the neurons will fire and the response without output noise will be a vector of all zeros. Thus, the decoded stimulus will be completely random with respect to the original stimulus.
- For an $O < K$ code, only subpopulations that do not represent the bit-flipped feature will be active. The code will operate as a code of the same order on a stimulus space with $K - 1$ features, and will have only $\frac{K-O}{K}$ of the representation energy of the original code. Thus, all codes will infer random values for the bit-flipped feature, and will encode the rest of the values according to a code of this nature, which will lead to reduced performance for higher order codes (though this reduction is partly corrected by the greater reliability of those codes as in Fig S3D).

2. With probability $1 - \frac{1}{n}$, the bit-flip will change a 0 to a 1 for one of the features. For all codes, this will result in a second codeword becoming equally likely in our decoder, and lead to a 50 % chance of error due to this input perturbation. It will also increase the representation energy used by the code.

In simulations of codes with $K = 3$ and $n = 5$, we see that the input bit-flip noise produces a base mean squared-error even without any output noise (Fig S3C), due to the effects described above. The $O = 1$ and $O = 2$ codes have equivalent performance, while the $O = 3 = K$ code performs worse (again, following the pattern described above). However, when we simulate the full channel over a variety of SNRs at a fixed input bit-flip probability (Fig S3D), code performance replicates the broad trends of our MSE analysis in the main text (Fig 3). In particular, the mixed codes show a faster decay of mean squared-error as SNR increases, but the full-order code decays to a larger mean squared-error baseline than either of the other codes. This baseline mean squared-error is entirely due to the input noise, and cannot be reduced by increase of the code SNR. As in the case without input noise, increasing the response field size (σ_{rf}) of the neurons in the code is likely to increase performance and correct some of the errors made due to input noise. The degree to which this changes performance will be explored in future research.

A.6 The rate-distortion bound and mutual information calculation

To calculate the rate-distortion bound (RDB) for our source distribution, we use a Python implementation of the iterative Blahut-Arimoto algorithm [5, 6]. Since the optimization problem is convex, the algorithm is guaranteed to converge on the right solution, given enough iterations. To ensure an adequate number of iterations, we terminate the algorithm only when successive steps are less than 10^{-10} change in error probability magnitude.

To evaluate our codes alongside the RDB, we must calculate the mutual information between the stimulus distribution X and the distribution of our stimulus estimates $\hat{X}$. So,

$$I(X; \hat{X}) = H(\hat{X}) - H(\hat{X}|X) \tag{S.31}$$

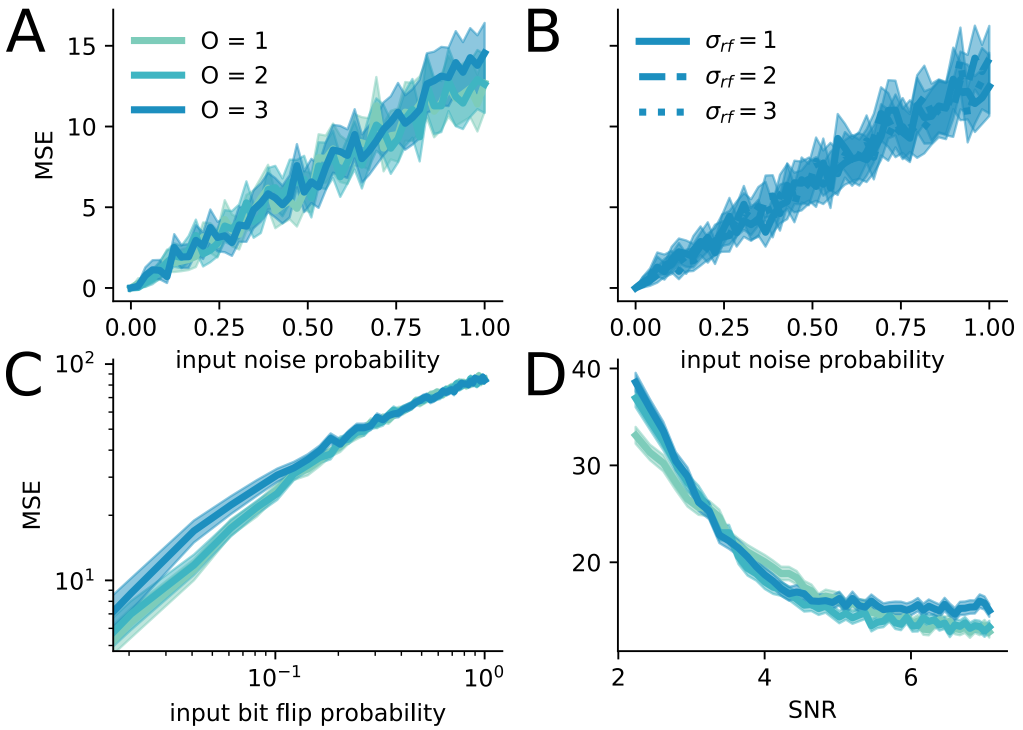

Fig S3. Code order does not have an effect on sensitivity to local input noise, related to Fig 2. For all panels, $K = 3$, $n = 10$. **A** The mean squared-error (MSE) of different codes as a function of input noise without output noise, represented as the probability of each feature taking on the value above or below its "true" value. **B** The same as **A** but for the $O = 3$ code with different RF sizes. **C** An additional simulation with non-local input noise – where bits in an input $O = 1$ code are randomly flipped with the probability given on the x-axis. The error rate of the resulting $O = 1, 2, 3$ codes with the same parameters as above is plotted. **D** A simulation with non-local input noise and output noise. The result here is similar to that without input noise in Fig 3, except that the $O = 3$ code has a higher error rate at high SNR due to its increased sensitivity to input noise, shown in **C**.

where

$$H(Y) = -\sum_{y \in Y} P(y) \log_2 P(y) \tag{S.32}$$

$$H(Y|Z) = -\sum_{z \in Z} P(z) \sum_{y \in Y} P(y|z) \log_2 P(y|z) \tag{S.33}$$

$$= \sum_{z \in Z} P(z) H(Y|Z = z) \tag{S.34}$$

To compute these quantities, we rely the observation that $P(X) = P(\hat{X})$. That is, both distributions are uniform, with $P(\hat{x}) = P(x) = \frac{1}{n^K}$. This can be seen from the fact that none of our codewords have more (or fewer) neighbors at any given distance than any of our other codewords (see statement 4).

Using this,

$$I(X;\hat{X}) = H(\hat{X}) - H(\hat{X}|X) \tag{S.35}$$
$$= H(X) - H(\hat{X}|X) \tag{S.36}$$
$$= K\log_2 n - \sum_{x\in X} P(x)H(\hat{X}|X=x) \tag{S.37}$$

Since $P(x) = \frac{1}{n^K}$ and $P(\hat{X}|X=x)$ has the same entropy for all x, following from the observation above, it is enough to estimate

$$I(X;\hat{X}) = K\log_2 n - H(\hat{X}|X=x) \tag{S.38}$$

for a particular x. We do this via numerical simulations (see Full channel details in Methods for details).

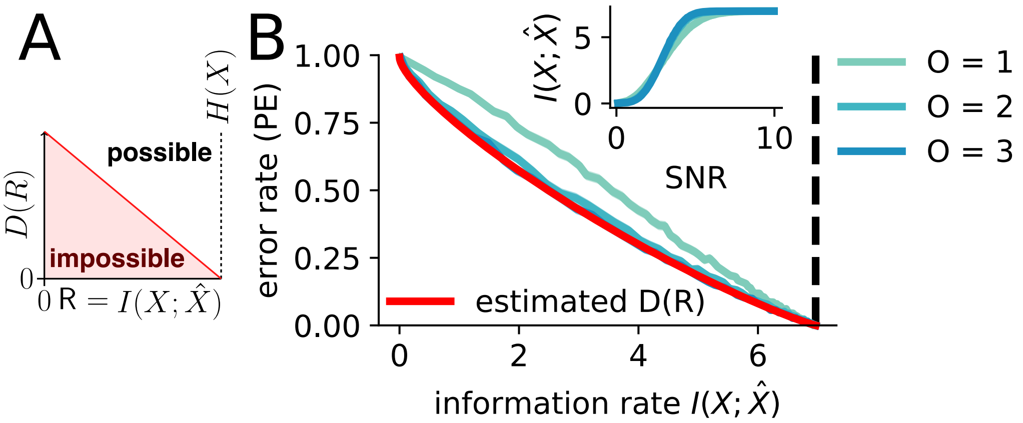

Fig S4. The mixed codes come close to or achieve the rate-distortion bound while the pure code does not, related to Fig 2. **A** A schematic of the rate-distortion bound. The bound is a function on the information rate-error rate plane dividing a region of possible codes from a region of impossible codes. The bound depends only on the stimulus distribution and distortion type, it does not depend on any code properties. Thus, we evaluate codes relative to the bound. If a code achieves the bound, that means it achieves the most efficient possible mapping from stimulus information to distortion – i.e., it uses the fewest possible bits to achieve a particular error rate. The rate-distortion bound goes to zero as $I(X;\hat{X})$ approaches $H(X)$ since the mutual information between the stimulus and its estimate cannot exceed the entropy of the stimulus. **B** For $K = 3$, $n = 5$ and a uniform probability distribution over the stimuli, we evaluated codes with different levels of mixing relative to the rate-distortion bound (red). We show that the two mixed codes $O = 2$ and $O = 3$ achieve or come close to achieving the rate-distortion bound, while the pure code does not. (inset) The transformation from SNR to $I(X;\hat{X})$ for each of the codes is fairly similar, though the mixed codes are slightly less efficient at low SNR and slightly more efficient at high SNR.

A.7 Representation energy required to reach a .1% error rate

We also compared codes on the basis of how much representation energy they required reach a .1% error rate given a fixed noise variance. These results are given in Fig S5, for noise variance $\sigma^2 = 10$.

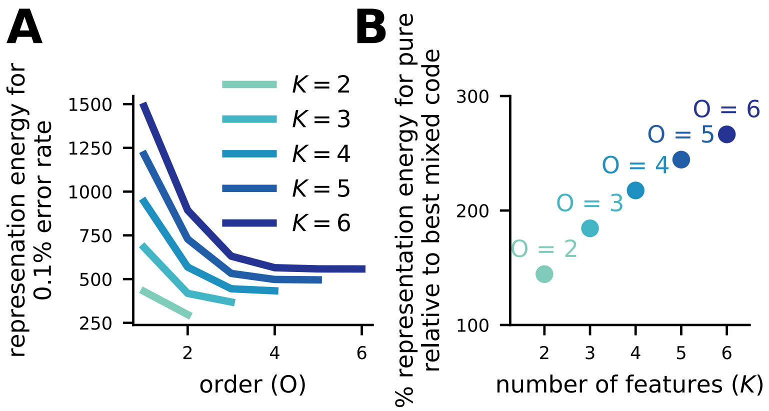

Fig S5. Mixed codes require less representation energy to achieve the same error rates as pure codes, related to Fig 2. For both plots, $n = 5$ and the noise variance $\sigma^2 = 10$. **A** The amount of representation energy required to reach a 1% error rate for codes of all orders given various numbers of features K. The code requiring the least energy is always the $O = K$ or $O = K - 1$ code. **B** The percent more representation energy required by the pure code to reach a 1% error rate compared to the optimal mixed code. The order of the optimal mixed code is indicated by the text above each marker.

A.8 Additional results on response fields

Generalizing our current framework to allow flexibly sized response fields (RFs) requires only a reformulation of the indicator function. Instead of performing an equality operation, it should instead perform a set membership operation, as

$$[i \in J] = \begin{cases} 0 & i \notin J \\ 1 & i \in J \end{cases} \tag{S.39}$$

where the set J is, in this case, a contiguous sequence of feature values of length σ_{rf}. Following this, for our main results, $\sigma_{\text{rf}} = 1$. Now, we explore how choosing $\sigma_{\text{rf}} > 1$ changes our results.

A.8.1 Effects on minimum distance, representation energy, and population size

Population size and representation energy change with RF size to ensure that full coverage of the stimulus set is maintained. To achieve this, we arrange the code dimensions in a series of σ_{rf} overlapping lattices, where each lattice has non-overlapping RFs in a grid pattern. This strategy is not guaranteed to be the most efficient tiling of the space, but it is simple to implement and analyze – and it approximately meets the theoretical estimate of the dimensionality of the most efficient tiling [53]. This RF tiling does, however, cause the stimuli on the edge of stimulus space to behave different from the stimuli near the center. In particular, stimuli within the RF-width of the maximum or minimum feature value for one or more features will have fewer neighbors than other stimuli and will therefore have lower error probabilities than more central stimuli. Thus, for our simulations with $\sigma_{\text{rf}} > 1$, the fact that we sample stimuli uniformly rather than with some other distribution does have a mild effect on our results. However, since the number of edge stimuli is a feature of the stimulus space, not the code, the proportion of edge to non-edge stimuli is the same across codes, thus different codes do not benefit more from the sampling of additional edge stimuli.

The increase of σ_{rf} has the following effects on our three principle code metrics.

Dimensionality:

$$D_O = \binom{K}{O} \sigma_{\mathrm{rf}} \left(\frac{n}{\sigma_{\mathrm{rf}}} + 1 \right)^O \tag{S.40}$$

Power:

$$P_O = \binom{K}{O} \sigma_{\mathrm{rf}} \tag{S.41}$$

Minimum distance:

$$\Delta_O = \left[2 \binom{K-1}{O-1} \right]^{\frac{1}{2}} \tag{S.42}$$

Note that minimum distance is not affected.

A.8.2 The optimal σ_{rf} for a given total energy

For a fixed K, O, n, and E, we want to find the σ_{rf} that maximizes minimum distance. For $E = \epsilon V + D_O$, and using $\delta(K, O, \sigma_{\mathrm{rf}}, V)$ as an expression for minimum distance after application of β to produce a code with power V, we can write the problem as:

$$\begin{aligned} L &= \delta \left(K, O, \sigma_{\mathrm{rf}}, \frac{E - D_O}{\epsilon} \right)^2 && \text{(S.43)} \\ &= \frac{2O}{K\epsilon} \left(\frac{E - D_O}{\sigma_{\mathrm{rf}}} \right) && \text{(S.44)} \\ &= \frac{2O}{K\epsilon} \left[\frac{E}{\sigma_{\mathrm{rf}}} - \binom{K}{O} \left(\frac{n}{\sigma_{\mathrm{rf}}} + 1 \right)^O \right] && \text{(S.45)} \end{aligned}$$

and now, to find the maximum, we will take the derivative $\frac{\partial L}{\partial \sigma_{\mathrm{rf}}}$,

$$\begin{aligned} \frac{\partial L}{\partial \sigma_{\mathrm{rf}}} &= \frac{2O}{K\epsilon} \frac{\partial L}{\partial \sigma_{\mathrm{rf}}} \left[\frac{E}{\sigma_{\mathrm{rf}}} - \binom{K}{O} \left(\frac{n}{\sigma_{\mathrm{rf}}} + 1 \right)^O \right] && \text{(S.46)} \\ &= \frac{2O}{K\epsilon} \left[-\frac{E}{\sigma_{\mathrm{rf}}^2} + \binom{K}{O} O \left(\frac{n}{\sigma_{\mathrm{rf}}} + 1 \right)^{O-1} \frac{n}{\sigma_{\mathrm{rf}}^2} \right] && \text{(S.47)} \end{aligned}$$

and now setting the LHS to zero,

$$\frac{\partial L}{\partial \sigma_{\text{rf}}} = 0 = \frac{2O}{K\epsilon}\left[-\frac{E}{\sigma_{\text{rf}}^2} + \binom{K}{O} O \left(\frac{n}{\sigma_{\text{rf}}}+1\right)^{O-1}\frac{n}{\sigma_{\text{rf}}^2}\right] \tag{S.48}$$

$$E = \binom{K}{O} O \left(\frac{n}{\sigma_{\text{rf}}}+1\right)^{O-1} n \tag{S.49}$$

$$\frac{E}{\binom{K}{O}On} = \left(\frac{n}{\sigma_{\text{rf}}}+1\right)^{O-1} \tag{S.50}$$

$$\left(\frac{E}{\binom{K}{O}On}\right)^{\frac{1}{O-1}} = \frac{n}{\sigma_{\text{rf}}}+1 \tag{S.51}$$

$$\sigma_{\text{rf,opt}} = n\left[\left[\frac{E}{On\binom{K}{O}}\right]^{\frac{1}{O-1}} - 1\right]^{-1} \tag{S.52}$$

See Fig S6F for a plot of this function. This formalization does ignore benefits of $\sigma_{\text{rf,opt}} > 1$ for reducing the number of nearest neighbors of high order codes.

A.8.3 Effects on error distribution

Increasing RF size has the effect of pulling the distribution of squared-error distortion more concentrated toward zero, while increasing the overall probability of an error (see Fig 3D). The increase in overall probability of an error for fixed SNR can be understood by the expression for code power given above, where an increase in RF size increases the power consumption of the code without producing a change in minimum distance.

However, increasing RF size does produce a change in the number of codewords at minimum distance and at succeeding distances. To see this, we can focus on the $O = K$ case: with $\sigma_{\text{rf}} = 1$, we know that all other codewords are nearest neighbors to a given codeword (Eq (S.15)) because only one dimension is active for each codeword. If, instead, we have $\sigma_{\text{rf}} = 2$, we know that each RF has a volume of σ_{rf}^K feature values, but their intersection must be of size 1. Thus, either active RF can be changed to $\sigma_{\text{rf}}^K - 1$ different RFs to still form a valid codeword. Thus, the number of nearest neighbors is $2\left(2^K - 1\right)$. With $\sigma_{\text{rf}} = 2$, all stimuli except the nearest neighbors will be at the same, further distance.

A.9 Error-reduction by mixed selectivity in the continuous case

Here, we adopt continuous stimulus features and RFs to test how well the benefits of mixed codes generalize to the continuous case (also see [37] for a deeper investigation of the continuous case). In particular, with stimuli $x \in X$ composed of K features, $x_i \sim U(0, n_i)$. Instead of the flat, discrete RFs defined in Additional results on response fields in Text S1, we use Gaussian RFs,

$$r(x|\sigma_{\text{w}}, c) = \exp\left(-\frac{\sum_i^{D_O}(x_i - c_i)^2}{2\sigma_{\text{w}}^2}\right) \tag{S.53}$$

which are then scaled by the amplifying transform β as described in Linear transform (β) in Methods. The rest of the channel is identical to the channel described previously,

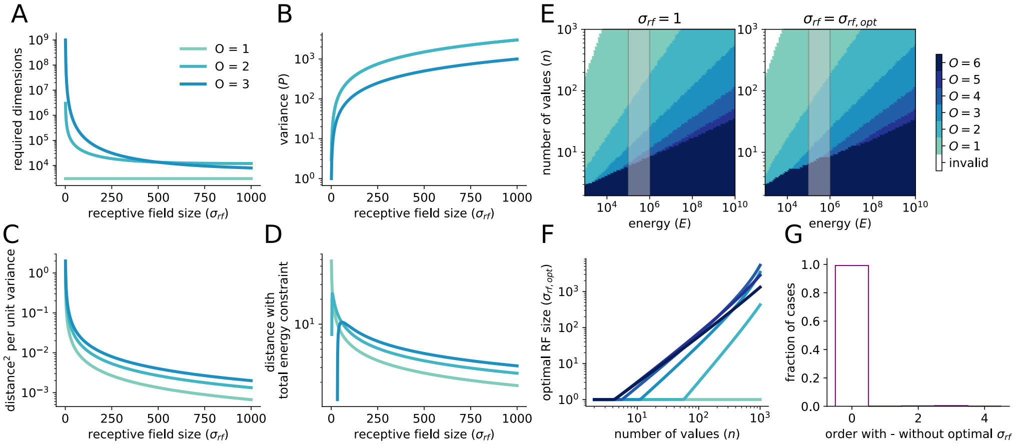

Fig S6. Changing response field (RF) size changes code properties, related to Fig 3. **a** The number of dimensions required to implement the code decreases by several orders of magnitude. **b** The power of the code increases by several orders of magnitude. **c** The tradeoff between minimum distance and code power remains constant if all codes are given the same RF size. **d** The RF size maximizing minimum distance under the total energy constraint differs between codes. **e** The code providing the highest minimum distance with $\sigma_{\mathrm{rf}} = 1$ (left) and $\sigma_{\mathrm{rf}} = \sigma_{\mathrm{rf,opt}}$ (right) as computed in Eq (S.52). They are only marginally different. **f** The optimal RF size for codes of different orders given features with different numbers of possible values. **g** Histogram of the differences in code order giving the highest distance from **e**.

including the additive noise. RFs are tiled in the same way, though now their width σ_{w} is independent of σ_{rf}, which dictates their tiling – as in Additional results on response fields in Text S1.

Our simulations show similar results to the discrete case (Fig S7), with higher order codes yielding lower MSE across all of the SNRs we investigated. Thus, the broad advantage of mixed codes apply in the continuous case as well. However, increasing RF size produces higher MSE, which is the opposite of our results in the discrete case. Future work is needed to discover why this is, and in what other ways the continuous case differs from the discrete case.

References

1. Atick JJ, Redlich AN. Towards a theory of early visual processing. Neural Computation. 1990;2(3):308–320.

2. Shannon CE. Probability of error for optimal codes in a Gaussian channel. Bell System Technical Journal. 1959;38(3):611–656.

3. Balasubramanian V, Berry MJ. A test of metabolically efficient coding in the retina. Network: Computation in Neural Systems. 2002;13(4):531–552.

4. Churchland MM, Yu BM, Cunningham JP, Sugrue LP, Cohen MR, Corrado GS, et al. Stimulus onset quenches neural variability: a widespread cortical phenomenon. Nature Neuroscience. 2010;13(3):369–378. doi:10.1038/nn.2501.

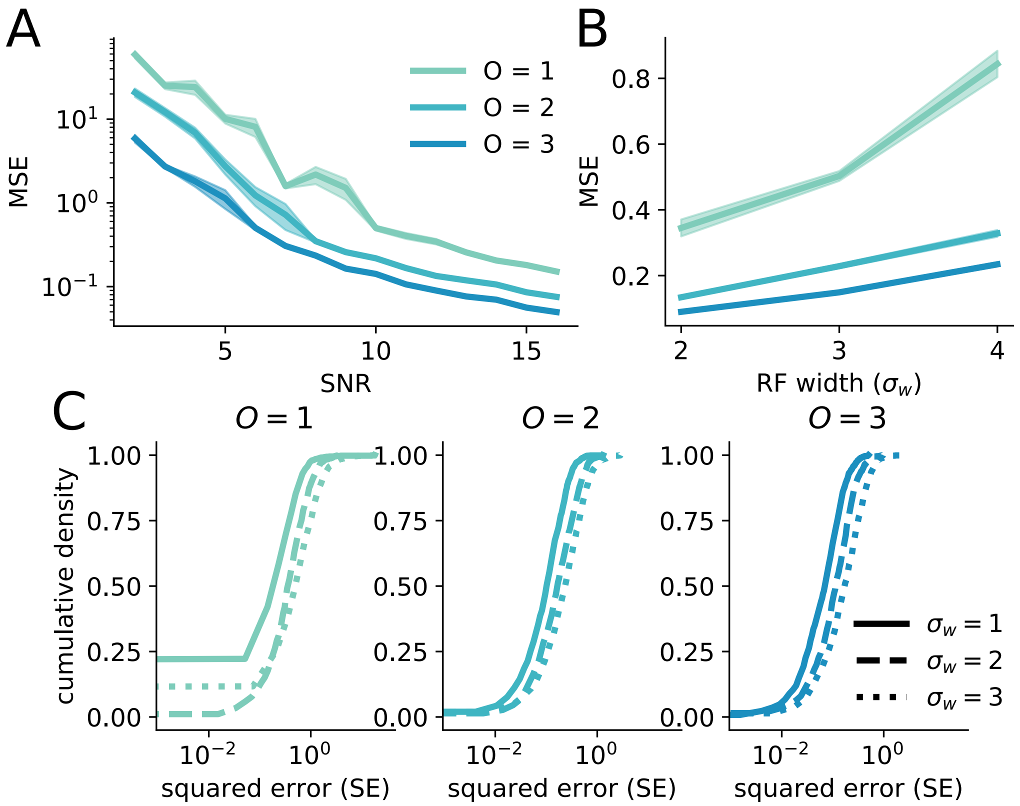

Fig S7. The benefits of mixed codes broadly generalize to continuous stimuli and RFs, related to Fig 3. **A** The MSE of codes of all orders with $K = 3$. The higher-order codes provide better performance than the lower-order codes. **B** MSE increases with RF size, which is contrary to the result in the discrete case (Fig 3d). **C** The cumulative distribution function of squared error for the three codes and for three different RF sizes.

5. Arimoto S. An algorithm for computing the capacity of arbitrary discrete memoryless channels. IEEE Transactions on Information Theory. 1972;18(1):14–20.

6. Blahut R. Computation of channel capacity and rate-distortion functions. IEEE transactions on Information Theory. 1972;18(4):460–473.
